# Development and Description of a National Cohort of Patients With Chronic Limb-Threatening Ischemia

**DOI:** 10.1016/j.jscai.2023.100982

**Published:** 2023-05-19

**Authors:** Alexander C. Fanaroff, Elias J. Dayoub, Lin Yang, Kaitlyn Shultz, Omar I. Ramadan, Elizabeth A. Genovese, Grace J. Wang, Scott M. Damrauer, Eric A. Secemsky, Sahil A. Parikh, Ashwin S. Nathan, Michael R. Jaff, Peter W. Groeneveld, Jay Giri

**Affiliations:** a Cardiovascular Medicine Division, Perelman School of Medicine, University of Pennsylvania, Philadelphia, Pennsylvania; b Penn Cardiovascular Outcomes, Quality, and Evaluative Research Center, University of Pennsylvania, Philadelphia, Pennsylvania; c Leonard Davis Institute for Health Economics, Philadelphia, Pennsylvania; d Penn Center for Health Incentives and Behavioral Economics, University of Pennsylvania, Philadelphia, Pennsylvania; e Division of Vascular Surgery and Endovascular Therapy, Perelman School of Medicine, University of Pennsylvania, Philadelphia, Pennsylvania; f Department of Genetics, Perelman School of Medicine, University of Pennsylvania, Philadelphia, Pennsylvania; g Corporal Michael J. Crescenz Veterans Affairs Medical Center, Philadelphia, Pennsylvania; h Smith Center for Cardiovascular Outcomes Research, Beth Israel Deaconess Medical Center, Harvard Medical School, Harvard University, Boston, Massachusetts; i Center for Interventional Cardiovascular Care, Division of Cardiology, Vagelos College of Physicians and Surgeons, Columbia University Irving Medical Center, New York, New York; j Boston Scientific Corporation, Marlborough, MA; k General Internal Medicine Division, Perelman School of Medicine, University of Pennsylvania, Philadelphia, Pennsylvania

**Keywords:** access and evaluation, critical limb ischemia, health care quality, peripheral artery disease

## Abstract

**Background:**

Chronic limb-threatening ischemia (CLTI) is a common condition with high rates of morbidity and mortality. Despite extensive literature documenting poor outcomes in patients with CLTI, as well as racial, ethnic, socioeconomic, and geographic disparities in these outcomes, process measures for high-quality CLTI care have not been developed. We developed the Chronic Limb threatening Ischemia Process PERformace (CLIPPER) cohort to develop and test the validity of CLTI care quality measures.

**Methods:**

Using inpatient and outpatient claims data from patients with fee-for-service Medicare from 2010 to 2019, we created a coding algorithm to identify patients with CLTI. To qualify for a CLTI diagnosis, patients had to have either diagnostic codes for peripheral artery disease and for ulceration, infection, or gangrene on the same inpatient or outpatient claim or a CLTI-specific diagnostic code. Patients were also required to have a procedural code indicating arterial vascular testing within 6 months before or after the earliest qualifying CLTI diagnostic code(s). We describe baseline characteristics and long-term outcomes of this cohort.

**Results:**

The final cohort comprised 1,130,065 patients diagnosed with CLTI between 2010 and 2019. Mean (±SD) age of the cohort was 75 ± 5.8 years; 48.4% were women, and 14.6% were Black. Within 30 days of CLTI diagnosis, 20.4% of patients underwent either percutaneous or surgical revascularization. Within 6 months, 3.3% of patients underwent major amputation; 16.7% of patients died within 1 year and 50.3% within 5 years.

**Conclusions:**

We described the development of a cohort of fee-for-service Medicare patients with CLTI using inpatient and outpatient Medicare claims data. CLIPPER will be a resource for developing a set of process measures that can be captured from administrative claims data, with plans to describe their association with limb outcomes and corresponding racial, ethnic, socioeconomic, sex-based, and geographic variability.

## Highlights

• Process measures for management of patients with chronic limb-threatening ischemia have not been developed.
• Inpatient and outpatient Medicare claims were used to identify patients with chronic limb-threatening ischemia.
• CLIPPER includes baseline data and outcomes from 1.13 million patients with chronic limb-threatening ischemia.
• Five-year mortality was 50%, revascularization 40%, and major amputation 7%.

## Central Illustration

**Figure:**
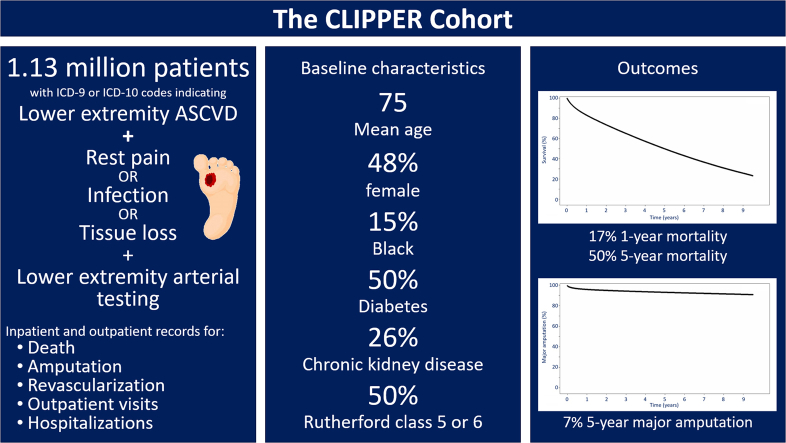

## Introduction

Peripheral artery disease (PAD), defined as partial or complete occlusion of at least 1 major lower extremity peripheral artery by atherosclerotic disease, affects 17% of patients aged 65 years or older.^1^^,^^2^ Patients with PAD may be asymptomatic, experience symptoms with ambulation, or develop chronic limb-threatening ischemia (CLTI), a condition characterized by rest pain, nonhealing wounds, and gangrene.^3^, ^4^, ^5^ Most studies describing the natural history of PAD indicate that 5% to 10% of patients with asymptomatic PAD progress to CLTI.^6^ Treatment for CLTI involves endovascular or surgical revascularization to improve limb perfusion, local wound care to control infection and improve wound healing, and guideline-directed medical therapy to reduce cardiovascular disease risk.^6^ Without prompt and aggressive treatment, >20% of patients with CLTI will undergo major lower extremity amputation within 12 months after diagnosis and >20% will die.^7^^,^^8^

For multiple common and morbid cardiovascular diseases—such as heart failure (HF), myocardial infarction (MI), and stroke—quality metrics have been derived from administrative data to measure processes of care (including time to appropriate revascularization), describe disparities in treatment and outcomes, create benchmarks by which individual hospitals and health systems can be evaluated, and catalyze local and national quality improvement efforts.^9^, ^10^, ^11^, ^12^, ^13^, ^14^ Like these conditions, CLTI is common, is highly morbid, and requires prompt treatment, yet no similar quality metrics have been developed to measure processes of care related to CLTI. Although numerous studies have described nationwide gaps in PAD diagnosis and management, as well as racial, geographic, and socioeconomic disparities in PAD outcomes,^15^, ^16^, ^17^, ^18^, ^19^, ^20^, ^21^, ^22^, ^23^, ^24^ little is understood about process measures underlying those gaps or hospital/health system variability in care processes or outcomes. Development and validation of a set of process measures related to CLTI management that can be obtained from Medicare claims data could facilitate large-scale quality improvement efforts to advance management of this highly morbid condition.

We therefore used Medicare fee-for-service Part A and B data to define the Chronic Limb threatening Ischemia Process PERformance cohort (CLIPPER), a cohort of patients with CLTI and longitudinal follow-up, including processes of care (outpatient visits, diagnostic imaging, inpatient admissions, and revascularization) and short-term and long-term outcomes. Within CLIPPER, we will study the association of process measures with outcomes, seeking to define process measures that can be used as quality indicators. In this manuscript, we describe how the cohort was developed, baseline characteristics of included patients, and short-term and long-term clinical outcomes.

## Methods

### Data sources

CLIPPER was created using Medicare fee-for-service claims data, including inpatient Medicare Provider Analysis and Review (MEDPAR) files derived from Part A claims, Part B carrier claims, enrollment information, vital status data, and chronic conditions data for 100% of patients with diagnostic codes consistent with CLTI. The University of Pennsylvania institutional review board designated this study as exempt from review. Analyses were conducted using SAS 9.4 (SAS Institute).

### Study cohort, inclusion criteria, and exclusion criteria

CLIPPER consists of patients aged between 66 years or older and 86 years or younger at the time of a CLTI diagnosis between July 1, 2010, and December 31, 2019. The lower age limit was selected to ensure that all patients had at least 6 months of data to identify comorbidities before the initial CLTI diagnosis, as Medicare becomes universal at the age of 65 years. The upper age limit was selected to exclude very old patients in whom percutaneous or surgical revascularization—the key therapeutic intervention in CLTI—might be contraindicated due to frailty or otherwise limited life expectancy. Patients with CLTI were identified using International Classification of Diseases, Ninth Edition (ICD-9) codes (for CLTI episodes on or before September 30, 2015) and tenth Edition (ICD-10) codes (for CLTI episodes after September 30, 2015) according to the algorithm outlined in Table 1. This coding algorithm was generated by manual review of ICD-9 and ICD-10 codes along with literature review.^25^^,^^26^ It was largely based on a similar, previously published algorithm using ICD-9 codes only, which had 75% sensitivity compared with a gold standard of chart review, and a κ value of 0.80.^26^ We translated this coding schema to ICD-10 codes. Broadly, included patients had to have (1) a diagnostic code indicating PAD and a diagnostic code indicating ulceration or infection on the same episode of care or (2) a diagnostic code specific for CLTI. Because CLTI requires anatomic confirmation of obstructive lower extremity arterial disease and diagnostic codes are occasionally used by clinicians before diagnostic confirmation, we restricted the cohort to patients who had actually undergone testing for PAD within 6 months before or 6 months after they otherwise met criteria for CLTI by the diagnostic coding algorithm. Testing for PAD was defined using Current Procedural Terminology (CPT) codes for ankle-brachial index measurement, computed tomography angiography, magnetic resonance angiography, arterial duplex ultrasonography, invasive angiography, or endovascular revascularization (Table 2). Endovascular revascularization was included as a test for PAD because some patients may have undergone diagnostic angiography followed by ad hoc revascularization, and administrative claims may not have captured the diagnostic angiography procedure. When describing the proportion of patients undergoing each testing modality, patients who were included based only on a code for endovascular revascularization were counted as having undergone diagnostic angiography.

**Table 1:** Coding algorithm for identifying patients with CLTI.

	Codes indicating peripheral artery disease		Codes indicating ulcer or infection		Codes specific for CLTI	
ICD-9 codes (before September 30, 2015)	250.7x, 249.70, 249.71	Diabetes with peripheral circulatory disorders	707.1x	Lower extremity ulcer, except decubitus	440.22	Lower extremity atherosclerosis with rest pain
	440.20, 440.21, 440.29	Lower extremity atherosclerosis	730.0x, 730.1x, 730.2x	Osteomyelitis	440.23	Lower extremity atherosclerosis with ulceration
	–	–	785.4	Gangrene	440.24	Lower extremity atherosclerosis with gangrene
	–	–	682.6, 682.7, 681.1	Lower extremity cellulitis	–	–
ICD-10 codes (after September 30, 2015)	I70.21x, I70.29x, I70.20x	Lower extremity atherosclerosis	M86.1x, M86.2x, M86.3x, M86.4x, M86.6x, M86.7x, M86.8x, M86.9	Osteomyelitis	I70.22x	Lower extremity atherosclerosis with rest pain
	I73.9	Peripheral vascular disease	I96	Gangrene	I70.23x, I70.24x, I70.25x	Lower extremity atherosclerosis with ulceration
	–	–	L97.1x, L97.2x L97.3x, L97.4x, L97.5x, L97.6x, L97.8x, L97.9x,	Lower extremity ulcer, except decubitus	I70.26x	Lower extremity atherosclerosis with gangrene
	–	–	E08.621, E09.621, E10.621, E11.621, E13.621, E09.622, E10.622, E11.622, E13.622	Diabetes with ulcer	E08.52, E09.52, E10.52, E11.52, E13.52	Diabetes with peripheral angiopathy and gangrene
	–	–	L03.03x, L03.115, L03.116, L03.119	Lower extremity cellulitis	–	–

For a diagnosis of CLTI, patients had to have either 1 diagnosis indicating lower extremity peripheral artery disease and 1 indicating ulceration or infection in the same claim (MEDPAR or Part B) or a specific CLTI code.

CLTI, chronic limb-threatening ischemia.

**Table 2:** CPT codes indicating testing for peripheral artery disease.

Testing modality	CPT codes
Ankle-brachial index	93922, 93923, 93924
Arterial duplex ultrasound	93925, 93926
Computed tomographic angiography	75635, 73706, 72191
Invasive angiography	36200, 36245, 36246, 36247, 36248, 75625, 75710, 75716, 75736
Magnetic resonance angiography	73725, 72198
Endovascular revascularization	37220, 37221, 37222, 37223, 37224, 37225, 37226, 37227, 37228, 37229, 37230, 37231, 37232, 37233, 37234, 37235

CPT, Current Procedural Terminology.

Rutherford classifications were assigned to each patient based on a coding algorithm applied to the initial episode of care in which patients entered the cohort. The presence of a code for gangrene indicated Rutherford Class 6. The presence of a code for osteomyelitis or ulcer indicated Rutherford Class 5. All other patients were assigned to Rutherford Class 4.

### Outcomes

Outcomes evaluated in CLIPPER include all-cause mortality, major lower extremity amputation, minor lower extremity amputation, percutaneous lower extremity revascularization, surgical lower extremity revascularization, and all-cause hospital admission. All-cause mortality was ascertained from Medicare denominator files. All procedural outcomes were ascertained from ICD-9, ICD-10, or CPT codes. Supplemental Table S1 includes all codes used to identify these outcomes.

### Statistical analysis

We describe baseline demographic and clinical characteristics of the study sample with continuous variables presented as mean with SD or median with IQR and categorical variables are presented as count with proportion. We created Kaplan-Meier and cumulative incidence curves, as appropriate, for all-cause mortality, lower extremity revascularization (percutaneous or surgical), and major lower extremity amputation over the 10-year follow-up. We also stratified by Rutherford classes to evaluate whether clinical severity of CLTI as defined by our algorithm correlated with worse outcomes. In addition to curves created for the overall cohort, we also created separate curves for patients with single CLTI-specific ICD-9 or ICD-10 codes (ICD-9: 440.22, 440.23, or 440.24; ICD-10: I70.22x, I70.23x, I70.24x, I70.25x, I70.26x, E08.52, E09.52, E10.52, E11.52, or E13.52) as a sensitivity analysis. We also describe baseline characteristics and outcomes of patients excluded from the sample due to a lack of lower extremity arterial testing.

## Results

**Figure 1:**
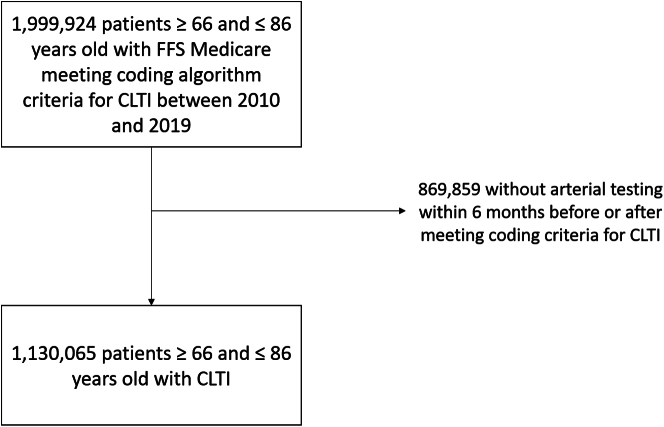
**Flow diagram of patient cohort included in the study.** The CLIPPER cohort consisted of patients aged between 66 and 86 years who had fee-for-service (FFS) Medicare, inpatient or outpatient billing codes indicating CLTI, and at least 1 episode of arterial testing within the 6 months before or after the episode meeting coding criteria for CLTI. CLTI, chronic limb-threatening ischemia.

Overall, 1,999,924 patients aged between 66 and 86 years met criteria for CLTI by virtue of our coding algorithm (1 code for PAD plus 1 code for ulceration or infection in the same episode of care, or 1 CLTI-specific code) between 2010 and 2019. Of these patients, 869,859 did not undergo lower extremity arterial testing in the 6 months before or 6 months after they otherwise met criteria for CLTI by administrative claims and were excluded (Figure 1), leaving a final analytic cohort of 1,130,065 patients. Baseline characteristics and outcomes of patients who were excluded due to lack of lower extremity arterial testing are presented in Supplemental Table S2 and Supplemental Figure S1. Compared with patents included in the cohort, these patients were older, had fewer cardiovascular and noncardiovascular comorbidities, and had a substantially lower incidence of revascularization and major amputation over short-term and long-term follow-ups.

The proportion of patients meeting each qualifying diagnostic code is shown in Supplemental Table S3. Of 807,217 patients included into the cohort during the ICD-9 era, 453,565 (56.2%) presented with 1 of the 3 CLTI-specific codes; 282,426 (61.4%) of the 460,042 patients included into the cohort during the ICD-10 era presented with 1 of the 10 CLTI-specific codes.

**Figure 2:**
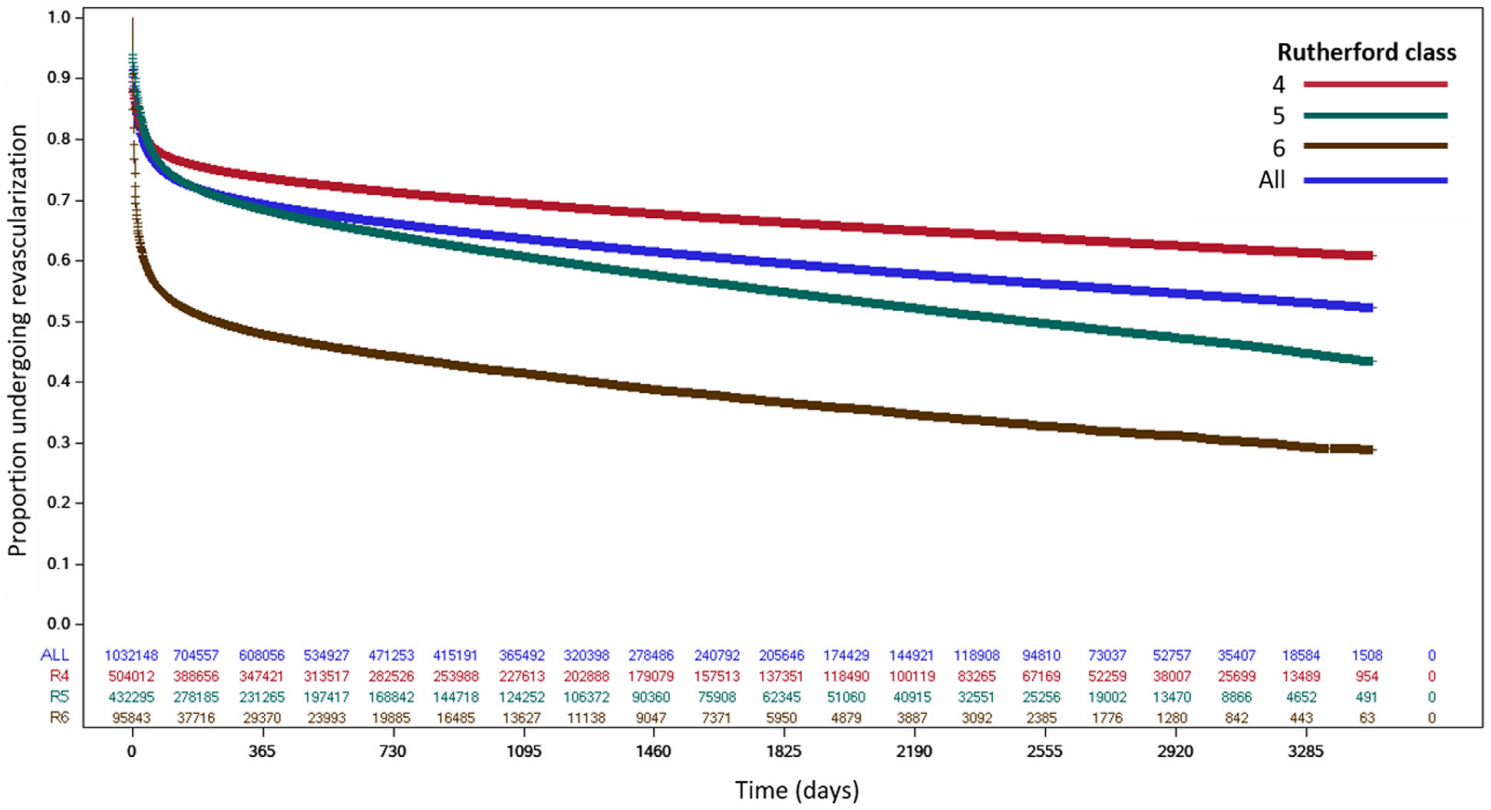
**Incidence of lower extremity revascularization in the study cohort overall and by Rutherford class.** The incidence of percutaneous or surgical lower extremity revascularization was highest immediately after CLTI diagnosis, with 20.4% of patients undergoing revascularization within 30 days and 28.0% within 6 months. CLTI, chronic limb-threatening ischemia.

Patients in the cohort had a mean (±SD) age of 75.3 ± 5.8 years and 48.4% (n = 546,480) were women; 77.4% (n = 874,798) were white and 14.6% (n = 165,431) were Black. Categorized by Rutherford class at the time of the initial CLTI diagnosis, 49.9% (n = 564,155) were Rutherford 4, 40.7% (n = 460,120) Rutherford 5, and 9.4% (n = 105,790) Rutherford 6. The mean Elixhauser comorbidity score was 5.5 ± 3.1, and 81.5% (n = 921,106) of patients had hypertension, 50.0% (n = 565,316) diabetes mellitus, and 26.0% (n = 293,744) HF (Table 3). With respect to diagnostic testing within the 6 months before or after entry into the cohort, 69.1% (n = 780,750) underwent ankle-brachial index testing, 53.8% (n = 607,844) duplex ultrasonography, and 32.7% (n = 370,558) invasive angiography; 48.4% (n = 546,819) of patients underwent more than one imaging test. Within 30 days of CLTI diagnosis, 20.4% of patients underwent either percutaneous or surgical revascularization, of which 28.0% was within 6 months (Figure 2). Patients with Rutherford class 4 were least likely to undergo revascularization, whereas those with Rutherford classes 5 and 6 were more likely to.

**Table 3:** Baseline characteristics and arterial testing of the overall cohort.

Variable	Total (N = 1,130,065)
Baseline characteristics	
Age, y	75.3 ± 5.8
Female sex	546,480 (48.4)
Race/ethnicity	
White	874,798 (77.4)
Black	165,431 (14.6)
Asian	22,646 (2.0)
Hispanic	34,042 (3.0)
Native American	6,570 (0.6)
Other/unknown	26,578 (2.4)
Region	
Midwest	232,658 (20.6)
Northeast	246,788 (21.8)
South	467,127 (41.3)
West	179,001 (15.8)
Rutherford class	
4	564,155 (49.9)
5	460,120 (40.7)
6	105,790 (9.4)
Elixhauser comorbidities	5.5 ± 3.1
Congestive heart failure	293,744 (26.0)
Valvular heart disease	203,107 (18.0)
Diabetes mellitus	565,316 (50.0)
Hypertension	921,106 (81.5)
Chronic kidney disease	297,198 (26.3)
Chronic lung disease	326,160 (28.9)
Cardiac arrhythmia	82,696 (7.3)
Obesity	164,643 (14.6)
Depression	151,314 (13.4)
Imaging tests within 6 mo before or after CLTI diagnosis	
Ankle-brachial index	780,750 (69.1)
Computed tomography angiography	142,244 (12.6)
Invasive angiography	370,558 (32.7)
Magnetic resonance angiography	15,783 (1.4)
Duplex ultrasound	607,844 (53.8)
More than 1 test	546,819 (48.4)

Values are n (%) unless or mean ± SD.

CLTI, chronic limb-threatening ischemia.

**Figure 3:**
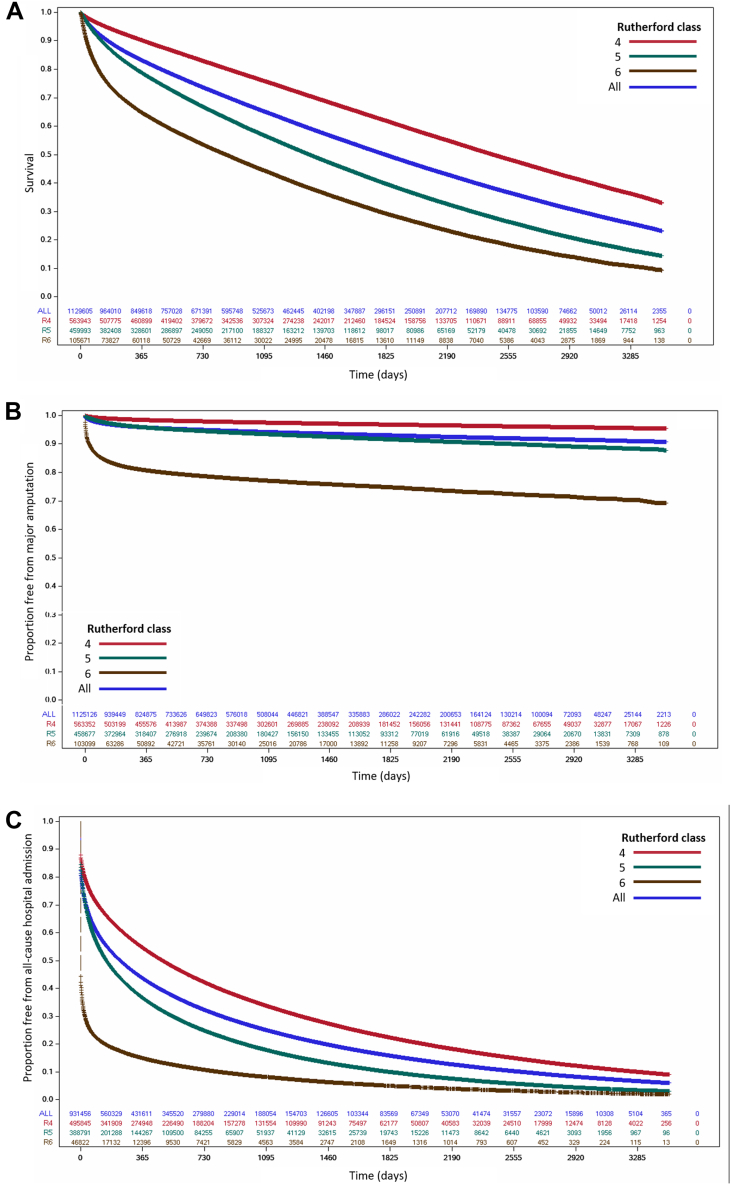
**Incidence of all-cause death, major amputation, and all-cause hospital admission in the study cohort overall and by Rutherford class.** The cumulative incidence of (**A**) all-cause mortality; (**B**) major amputation, and (**C**) all-cause hospital admission. Over 1-year follow-up, 16.7% of the patients died; >50% died through 5 years and >75% through 10 years. Within 6 months of CLTI diagnosis, 3.3% of the patients underwent major lower extremity amputation; the rate slowed down thereafter but 0.5% to 1.0% of the surviving patients continued to undergo major lower extremity amputation each year over long-term follow-up. Over 1-year follow-up, >50% of the patients were admitted to the hospital, and ∼90% were admitted at least once over the course of long-term follow-up. CLTI, chronic limb-threatening ischemia.

Over long-term follow-up, 16.7% of the patients died by 1-year follow-up, 35.1% by 3-year follow-up, 50.3% by 5-year follow-up, and 76.5% by 10-year follow-up (Figure 3). Risk of major amputation was highest in the 6 months following CLTI diagnosis, with 3.3% of patients undergoing major amputation in this time frame. Approximately 1% of surviving patients underwent major amputation between 6 and 12 months’ follow-up, and major amputations continued to accumulate at a rate of approximately 0.5% to 1.0% per year over long-term follow-up. The risk of all-cause death and major amputation was highest for patients with Rutherford class 6, followed by Rutherford class 5 and Rutherford class 4. Similarly, >50% of the patients were admitted to the hospital through the 1-year follow-up, with higher rates in patients with Rutherford classes 5 and 6 than those with Rutherford class 4. Event rates for patients with one of the CLTI-specific diagnostic codes were similar to those in the entire cohort (Supplemental Figure S2).

## Discussion

In this article, we described the creation of CLIPPER, a cohort of 1.13 million patients aged between 66 and 86 years with fee-for-service Medicare diagnosed with CLTI between 2010 and 2019 (Central Illustration). Through an extensive manual review of ICD-9 and ICD-10 codes and review of existing literature, we developed a novel process for comprehensively identifying patients with CLTI in both inpatient and outpatient settings using inpatient and outpatient Medicare claims data, supplemented with procedure codes for arterial testing required to diagnose CLTI. Consistent with what is known about patients with CLTI, CLIPPER consists of older individuals with multiple comorbidities, and Black patients are overrepresented in comparison with the overall population of fee-for-service Medicare beneficiaries (14.6% vs 9.2%).^27^ Outcomes for patients in CLIPPER are poor, consistent with previously described outcomes for patients with CLTI. Given longitudinal follow-up and the opportunity for statistical adjustment using methods appropriate for administrative claims data, this data set can be used to describe associations between process measures related to CLTI care and outcomes and to describe existing racial, ethnic, socioeconomic, sex-based, and geographic disparities in delivery of quality CLTI care on a national scale.

**Central Illustration:**
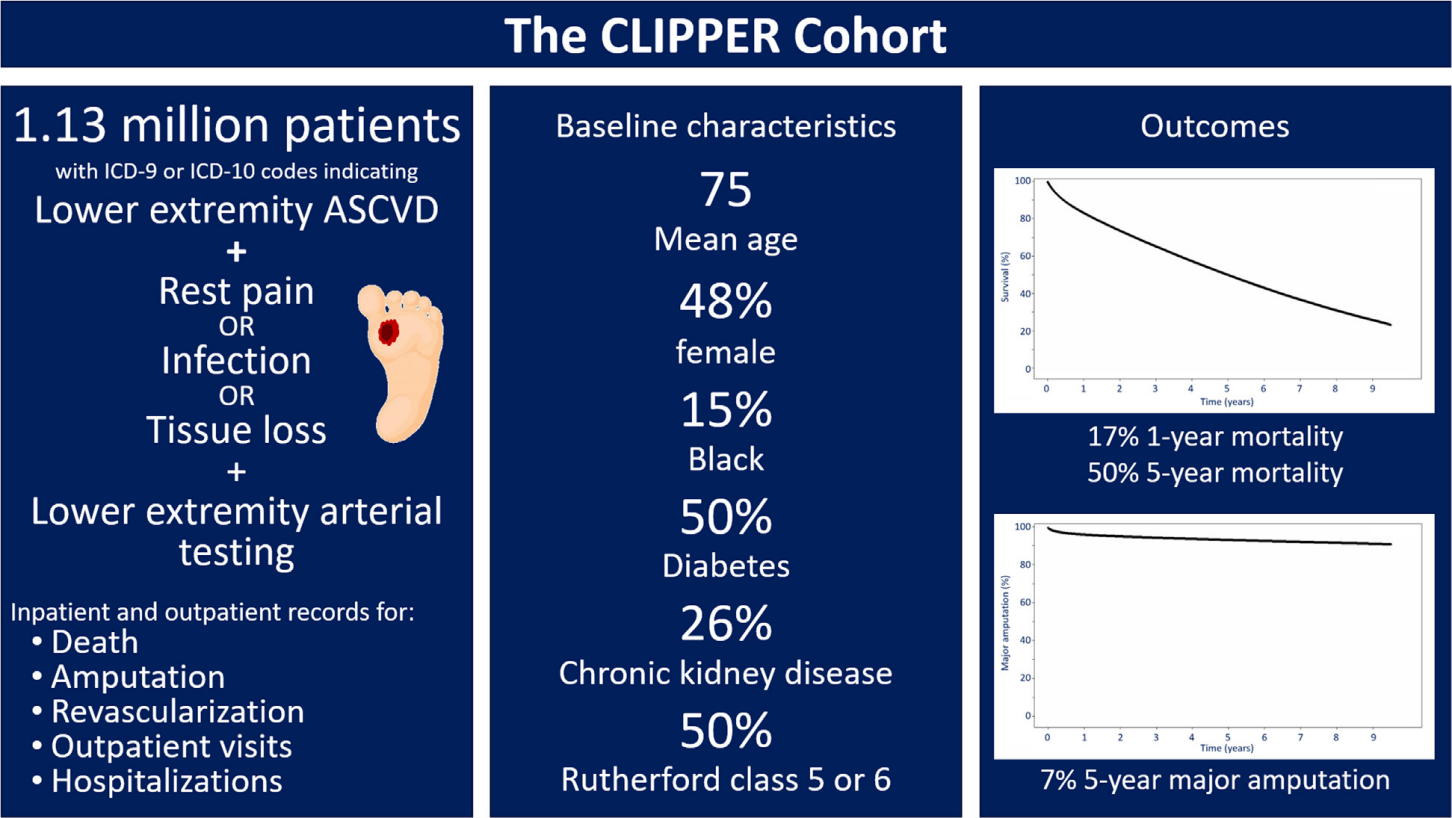
**Development of the CLIPPER cohort.** CLIPPER includes 1.13 million patients with CLTI as defined by ICD-9 and ICD-10 codes, plus a code for lower extremity arterial testing. Use of inpatient and outpatient claims allows description of baseline characteristics, details of hospitalizations and outpatient visits, and outcomes including death, revascularization, and major amputation. CLTI, chronic limb-threatening ischemia; ICD, International Classification of Diseases.

Process and outcome measures captured from administrative claims data and registries have been used as a key indicator of quality care in highly morbid cardiovascular conditions such as HF, MI, and stroke.^9^, ^10^, ^11^, ^12^, ^13^, ^14^ Hospital-level risk-adjusted mortality and readmission rates for these conditions are publicly reported by the Centers for Medicare and Medicaid Services (CMS), and process measures like door-to-balloon and door-to-needle times and times from presentation to electrocardiogram and brain imaging are reported by CMS and others. Although the modern quality assessment and improvement regime is not without its flaws and critics,^28^ foundational efforts to measure processes and outcomes have had wide-ranging impact on stroke, MI, and HF care.^29^, ^30^, ^31^ CLTI has a similar prognosis to these 3 cardiovascular conditions. In our study, short-term and long-term mortality after CLTI diagnosis was comparable with that after HF hospitalization, stroke, and MI, with 15% to 20% of the patients dying within 1 year and 50% of the patients dying within 5 years after diagnosis.^32^, ^33^, ^34^ Although CLTI is not as common as HF, MI, or stroke, there were ∼100,000 patients diagnosed per year in CLIPPER, which focused on a population of elderly individuals with traditional Medicare coverage. Despite the morbidity of CLTI and its prevalence, there has been no similar, widespread effort to capture process measures and outcomes to improve quality of care for CLTI. Ongoing, laudable efforts to measure the quality of lower extremity vascular care, such as the Vascular Quality Initiative and the National Cardiovascular Data Registry’s Peripheral Vascular Intervention Registry, have included only patients undergoing lower extremity revascularization,^35^^,^^36^ which our study shows is a minority of the overall CLTI population. Furthermore, procedure-focused registries exclude patients who may be too frail or high-risk to undergo procedures, introducing selection bias and limiting the ability of procedure-based registries to accurately assess disease-specific quality and outcomes.^37^ In addition, CLIPPER allows decade-long longitudinal assessment of both contact with the health care system in the form of visits, admissions, diagnostic testing, or procedures. Such longitudinal, disease-focused follow-up is more difficult to obtain in procedure-specific registries.

Lack of data describing quality of care and outcomes for patients with CLTI is partially attributable to the logistical challenges in capturing patients with CLTI. Unlike HF hospitalization, MI, and stroke, CLTI is often managed in the outpatient setting,^38^ and capturing the full cohort of patients with incident CLTI diagnoses necessarily requires the use of outpatient billing codes. In CLIPPER, we used Medicare Part A and B claims, enabling us to identify patients with CLTI whether they present to an outpatient clinic, outpatient-based catheterization laboratories, or ambulatory surgical centers or for inpatient admission. CLTI presents heterogeneously from a symptom and anatomic standpoint, and billing codes reflect this heterogeneity, challenging attempts to algorithmically identify patients with CLTI from administrative claims.^39^ Although we did not validate our coding scheme by a chart review, it has face validity for several reasons: (1) the required codes are logical extensions of the diagnostic process for capturing CLTI (presence of PAD plus evidence of rest pain, infection, ulceration, or gangrene); (2) the demographics and comorbidities of the patient population match those of previously described CLTI cohorts; (3) mortality is similar to that described in other CLTI cohorts; (4) revascularization, death, and amputation were more common in patients with more severe (higher Rutherford class) CLTI; and 5) patients had claims submitted for diagnostic testing appropriate for CLTI diagnosis confirmation contemporaneous with their entry into the cohort.^7^^,^^8^ Notably, the incidence of major amputation and revascularization are lower in CLIPPER compared with some other reports;^5^ however, most of these reports included patients diagnosed only in the inpatient setting or who underwent a revascularization procedure. The incidences of revascularization and amputation in CLIPPER are comparable with other cohorts that included an all-comers CLTI population, including those who did not undergo intervention and/or were not diagnosed while hospitalized.^40^^,^^41^

Using the nationwide data in CLIPPER, future studies will investigate the association between process measures related to CLTI care and outcomes—such as time from CLTI diagnosis to attempt at revascularization, receipt of wound care and other specialty vascular care, and use of diagnostic imaging or attempt at revascularization before amputation. We will also investigate racial, ethnic, sex-based, geographic, and between-hospital variation in these process measures. Disparities in PAD and CLTI outcomes have been widely reported,^19^, ^20^, ^21^^,^^23^^,^^42^, ^43^, ^44^, ^45^ but the extent to which process measures contribute to, or can better describe, these disparities have not. Better understanding of the association between these process measures and outcomes would have important implications for improving CLTI care and reducing disparities by allowing health systems to focus quality improvement efforts on these process measures. Because these process measures can be obtained from publicly available data, they could also serve as a template for national quality assessment and improvement programs.

### Limitations

The coding scheme we use to identify patients with CLTI has not been validated by chart review. However, our algorithms build on prior PAD work with ICD-9 codes that have been validated by chart review.^26^ Moreover, the demographics and outcomes of CLIPPER are reasonable for this patient population. Second, CLTI is a heterogeneous disease process, which is reflected in the different outcomes for patients with Rutherford 4, 5, and 6 disease. When developing process measures for the care of patients with CLTI, it may be important to consider this heterogeneity. Third, patients with Medicare Advantage—who comprise an increasing proportion of Medicare beneficiaries—were not included in this cohort, but the majority of Medicare beneficiaries during the study period had fee-for-service coverage and were eligible for inclusion in CLIPPER.^27^ Moreover, CLIPPER excludes patients younger than 65 and older than 86 years. Although CLIPPER's age range includes most patients with CLTI,^2^^,^^41^ it does exclude important subgroups of younger and older patients, and it will be important for future studies conducted using this cohort to clearly acknowledge the patients to which results do and do not apply. Lastly, administrative data lack rich clinical detail regarding severity of illness, anatomic factors, and detailed comorbidity assessments that may affect CLTI prognosis. We also do not have data on use of guideline-directed medical therapy, which is strongly associated with outcomes in this population.^46^ We were able to classify patients by Rutherford class, an important prognostic indicator. Although there are limitations related to the use of administrative data to assign Rutherford classification—for example, a patient with distal toe gangrene would be Rutherford class 6 in our coding schema but Rutherford class 5 using detailed clinical data—the strong observed association between Rutherford classes and outcomes indicates the broad effectiveness of our use of claims data to classify patients. Detailed clinical and anatomic detail are only available within the confines of chronic disease registries, which are necessarily limited to those hospitals with the financial and administrative capacity to participate. In the context of CLTI, the use of registries for quality improvement would exclude many vulnerable low-income patients without access to well-resourced hospitals with specialized vascular care,^16^^,^^45^^,^^47^ and would lead to considerable challenges obtaining longitudinal follow-up data. Therefore, use of administrative claims data—carefully analyzed and with limitations clearly delineated—is the best current solution for examining process measures for CLTI care.

## Conclusions

In this article, we describe the development of CLIPPER, a cohort of fee-for-service Medicare patients with CLTI captured in inpatient and outpatient Medicare claims data. Consistent with what is known about CLTI, the cohort consists of older adults with significant comorbidity burden, with Black individuals overrepresented. CLIPPER will be a resource for developing a set of process measures that can be obtained from administrative claims data and for describing their association with limb outcomes and racial, ethnic, sex-based, socioeconomic, and geographic variability.
