# Supplementary material for: Development and Description of a National Cohort of Patients With Chronic Limb-Threatening Ischemia
Source: J Soc Cardiovasc Angiogr Interv. 2023 May 19;2(4):100982. doi: 10.1016/j.jscai.2023.100982 (PMC11308495; doi:10.1016/j.jscai.2023.100982)

**SUPPLEMENTAL MATERIALS**

**Supplemental Table 1:** ICD-9, ICD-10, and CPT codes for revascularization and amputation

**Supplemental Table 2:** Baseline characteristics of patients excluded for lack of lower extremity arterial testing

**Supplemental Table 3:** Proportion of patients with each qualifying diagnosis code or combination of diagnostic codes

**Supplemental Figure 1**: Cumulative incidence of all-cause death (A), revascularization (B), and major amputation (C) overall and by Rutherford class among patients excluded for lack of lower extremity arterial testing

**Supplemental Figure 2:** Cumulative incidence of all-cause death (A), revascularization (B), and amputation (C) overall and by Rutherford class among patients with single CLTI-specific codes

**Supplemental Table 1:** ICD-9, ICD-10, and CPT codes for revascularization and amputation

| Outcomes | Codes |
| --- | --- |
| Major amputation | ICD-9: 8410, 8413, 8414, 8415, 8416, 8417, 843 |
|  | ICD-10: 0Y6M0Z0, 0Y6N0Z0, 0Y6C0Z1, 0Y6C0Z2, 0Y6C0Z3, 0Y6D0Z1, 0Y6D0Z2, 0Y6D0Z3, 0Y6H0Z1, 0Y6H0Z2, 0Y6H0Z3, 0Y6J0Z1, 0Y6J0Z2, 0Y6J0Z3, 0Y6F0ZZ, 0Y6G0ZZ, 0Y670ZZ, 0Y680ZZ, 0Y620ZZ, 0Y630ZZ, 0Y640ZZ |
|  | CPT: 27590, 27591, 27592, 27594, 27598, 27800, 27881, 27882, 27884, 27886, 27888, 27889, |
| Minor amputation | ICD-9: 8411, 8412 |
|  | ICD-10: 0Y6M0Z4, 0Y6M0Z5, 0Y6M0Z6, 0Y6M0Z7, 0Y6M0Z8, 0Y6M0Z9, 0Y6M0ZB, 0Y6M0ZC, 0Y6M0ZD, 0Y6M0ZF, 0Y6N0Z4, 0Y6N0Z5, 0Y6N0Z6, 0Y6N0Z7, 0Y6N0Z8, |
|  | CPT: 28120, 28122, 28124, 28220, 28225, 28800, 28805, 28810 |
| Endovascular revascularization | ICD-9: 3950, 3990, 0055, 1756 |
|  | ICD-10: 047C041, 047C046, 047C04Z, 047C056, 047C05Z, 047C066, 047C06Z, 047C076, 047C07Z, 047C0D1, 047C0D6, 047C0DZ, 047C0E6, 047C0EZ, 047C0F6, 047C0FZ, 047C0G6, 047C0GZ, 047C0Z1, 047C0Z6, 047C0ZZ, 047C341, 047C346, 047C34Z, 047C356, 047C35Z, 047C366, 047C36Z, 047C376, 047C37Z, 047C3D1, 047C3D6, 047C3DZ, 047C3E6, 047C3EZ, 047C3F6, 047C3FZ, 047C3G6, 047C3GZ,  047C3Z1, 047C3Z6, 047C3ZZ, 047C441, 047C446, 047C44Z, 047C456, 047C45Z, 047C466, 047C46Z, 047C476, 047C47Z, 047C4D1, 047C4D6, 047C4DZ, 047C4E6, 047C4EZ, 047C4F6, 047C4FZ, 047C4G6, 047C4GZ, 047C4Z1, 047C4Z6, 047C4ZZ, 047D041, 047D046, 047D04Z, 047D056, 047D05Z, 047D066, 047D06Z, 047D076, 047D07Z, 047D0D1, 047D0D6, 047D0DZ, 047D0E6, 047D0EZ, 047D0F6, 047D0FZ, 047D0G6, 047D0GZ, 047D0Z1, 047D0Z6, 047D0ZZ, 047D341, 047D346, 047D34Z, 047D356, 047D35Z, 047D366, 047D36Z, 047D376, 047D37Z, 047D3D1, 047D3D6, 047D3DZ, 047D3E6, 047D3EZ, 047D3F6, 047D3FZ, 047D3G6, 047D3GZ, 047D3Z1, 047D3Z6, 047D3ZZ, 047D441, 047D446, 047D44Z, 047D456, 047D45Z, 047D466, 047D46Z, 047D476, 047D47Z, 047D4D1, 047D4D6, 047D4DZ, 047D4E6, 047D4EZ, 047D4F6, 047D4FZ, 047D4G6, 047D4GZ, 047D4Z1, 047D4Z6, 047D4ZZ, 047E041, 047E046, 047E04Z, 047E056, 047E05Z, 047E066, 047E06Z, 047E076, 047E07Z, 047E0D1, 047E0D6, 047E0DZ, 047E0E6, 047E0EZ, 047E0F6, 047E0FZ, 047E0G6, 047E0GZ, 047E0Z1, 047E0Z6, 047E0ZZ, 047E341, 047E346, 047E34Z, 047E356, 047E35Z, 047E366, 047E36Z, 047E376, 047E37Z, 047E3D1, 047E3D6, 047E3DZ, 047E3E6, 047E3EZ, 047E3F6, 047E3FZ, 047E3G6, 047E3GZ, 047E3Z1, 047E3Z6, 047E3ZZ, 047E441, 047E446, 047E44Z, 047E456, 047E45Z, 047E466, 047E46Z, 047E476, 047E47Z, 047E4D1, 047E4D6, 047E4DZ, 047E4E6, 047E4EZ, 047E4F6, 047E4FZ, 047E4G6, 047E4GZ, 047E4Z1, 047E4Z6, 047E4ZZ, 047F041, 047F046, 047F04Z, 047F056, 047F05Z, 047F066, 047F06Z, 047F076, 047F07Z, 047F0D1, 047F0D6, 047F0DZ, 047F0E6, 047F0EZ, 047F0F6, 047F0FZ, 047F0G6, 047F0GZ, 047F0Z1, 047F0Z6, 047F0ZZ, 047F341, 047F346, 047F34Z, 047F356, 047F35Z, 047F366, 047F36Z, 047F376, 047F37Z, 047F3D1, 047F3D6, 047F3DZ, 047F3E6, 047F3EZ, 047F3F6, 047F3FZ, 047F3G6, 047F3GZ, 047F3Z1, 047F3Z6, 047F3ZZ, 047F441, 047F446, 047F44Z, 047F456, 047F45Z, 047F466, 047F46Z, 047F476, 047F47Z, 047F4D1, 047F4D6, 047F4DZ, 047F4E6, 047F4EZ, 047F4F6, 047F4FZ, 047F4G6, 047F4GZ, 047F4Z1, 047F4Z6, 047F4ZZ, 047H041, 047H046, 047H04Z, 047H056, 047H05Z, 047H066, 047H06Z, 047H076, 047H07Z, 047H0D1, 047H0D6, 047H0DZ, 047H0E6, 047H0EZ, 047H0F6, 047H0FZ, 047H0G6, 047H0GZ, 047H0Z1, 047H0Z6, 047H0ZZ, 047H341, 047H346, 047H34Z, 047H356, 047H35Z, 047H366, 047H36Z, 047H376, 047H37Z, 047H3D1, 047H3D6, 047H3DZ, 047H3E6, 047H3EZ, 047H3F6, 047H3FZ, 047H3G6, 047H3GZ, 047H3Z1, 047H3Z6, 047H3ZZ, 047H441, 047H446, 047H44Z, 047H456, 047H45Z, 047H466, 047H46Z, 047H476, 047H47Z, 047H4D1, 047H4D6, 047H4DZ, 047H4E6, 047H4EZ, 047H4F6, 047H4FZ, 047H4G6, 047H4GZ, 047H4Z1, 047H4Z6,  047H4ZZ, 047J041, 047J046, 047J04Z, 047J056, 047J05Z, 047J066, 047J06Z, 047J076, 047J07Z, 047J0D1, 047J0D6, 047J0DZ, 047J0E6, 047J0EZ, 047J0F6, 047J0FZ, 047J0G6, 047J0GZ, 047J0Z1, 047J0Z6, 047J0ZZ, 047J341, 047J346, 047J34Z, 047J356, 047J35Z, 047J366, 047J36Z, 047J376, 047J37Z, 047J3D1, 047J3D6, 047J3DZ, 047J3E6, 047J3EZ, 047J3F6, 047J3FZ, 047J3G6, 047J3GZ, 047J3Z1, 047J3Z6, 047J3ZZ, 047J441, 047J446, 047J44Z, 047J456, 047J45Z, 047J466, 047J46Z, 047J476, 047J47Z, 047J4D1, 047J4D6, 047J4DZ, 047J4E6, 047J4EZ, 047J4F6, 047J4FZ, 047J4G6, 047J4GZ, 047J4Z1, 047J4Z6, 047J4ZZ, 047K041, 047K046, 047K04Z, 047K056, 047K05Z, 047K066, 047K06Z, 047K076, 047K07Z, 047K0D1, 047K0D6, 047K0DZ, 047K0E6, 047K0EZ, 047K0F6, 047K0FZ, 047K0G6, 047K0GZ, 047K0Z1, 047K0Z6, 047K0ZZ, 047K341, 047K346, 047K34Z, 047K356, 047K35Z, 047K366, 047K36Z, 047K376, 047K37Z, 047K3D1, 047K3D6, 047K3DZ, 047K3E6, 047K3EZ, 047K3F6, 047K3FZ, 047K3G6, 047K3GZ, 047K3Z1, 047K3Z6, 047K3ZZ, 047K441, 047K446, 047K44Z, 047K456, 047K45Z, 047K466, 047K46Z, 047K476, 047K47Z, 047K4D1, 047K4D6, 047K4DZ, 047K4E6, 047K4EZ, 047K4F6, 047K4FZ, 047K4G6, 047K4GZ, 047K4Z1, 047K4Z6, 047K4ZZ, 047L041, 047L046, 047L04Z, 047L056, 047L05Z, 047L066, 047L06Z, 047L076, 047L07Z, 047L0D1, 047L0D6, 047L0DZ, 047L0E6, 047L0EZ, 047L0F6, 047L0FZ, 047L0G6, 047L0GZ, 047L0Z1, 047L0Z6, 047L0ZZ, 047L341, 047L346, 047L34Z, 047L356, 047L35Z, 047L366, 047L36Z, 047L376, 047L37Z, 047L3D1, 047L3D6, 047L3DZ, 047L3E6, 047L3EZ, 047L3F6, 047L3FZ, 047L3G6, 047L3GZ, 047L3Z1, 047L3Z6, 047L3ZZ, 047L441, 047L446, 047L44Z, 047L456, 047L45Z, 047L466, 047L46Z, 047L476, 047L47Z, 047L4D1, 047L4D6, 047L4DZ, 047L4E6, 047L4EZ, 047L4F6, 047L4FZ, 047L4G6, 047L4GZ, 047L4Z1, 047L4Z6, 047L4ZZ, 047M041, 047M046, 047M04Z, 047M056, 047M05Z, 047M066, 047M06Z, 047M076, 047M07Z, 047M0D1, 047M0D6, 047M0DZ, 047M0E6, 047M0EZ, 047M0F6, 047M0FZ, 047M0G6, 047M0GZ, 047M0Z1, 047M0Z6, 047M0ZZ, 047M341, 047M346, 047M34Z, 047M356, 047M35Z, 047M366, 047M36Z, 047M376, 047M37Z, 047M3D1, 047M3D6, 047M3DZ, 047M3E6, 047M3EZ, 047M3F6, 047M3FZ, 047M3G6, 047M3GZ, 047M3Z1, 047M3Z6, 047M3ZZ, 047M441, 047M446, 047M44Z, 047M456, 047M45Z, 047M466, 047M46Z, 047M476, 047M47Z, 047M4D1, 047M4D6, 047M4DZ, 047M4E6, 047M4EZ, 047M4F6, 047M4FZ, 047M4G6, 047M4GZ, 047M4Z1, 047M4Z6, 047M4ZZ, 047N041, 047N046, 047N04Z, 047N056, 047N05Z, 047N066, 047N06Z, 047N076, 047N07Z, 047N0D1, 047N0D6, 047N0DZ, 047N0E6, 047N0EZ, 047N0F6, 047N0FZ, 047N0G6, 047N0GZ, 047N0Z1, 047N0Z6,  047N0ZZ, 047N341, 047N346, 047N34Z, 047N356, 047N35Z, 047N366, 047N36Z, 047N376, 047N37Z, 047N3D1, 047N3D6, 047N3DZ, 047N3E6, 047N3EZ, 047N3F6, 047N3FZ, 047N3G6, 047N3GZ, 047N3Z1, 047N3Z6, 047N3ZZ, 047N441, 047N446, 047N44Z, 047N456, 047N45Z, 047N466, 047N46Z, 047N476, 047N47Z, 047N4D1, 047N4D6, 047N4DZ, 047N4E6, 047N4EZ, 047N4F6, 047N4FZ, 047N4G6, 047N4GZ, 047N4Z1, 047N4Z6, 047N4ZZ, 047P041, 047P046, 047P04Z, 047P056, 047P05Z, 047P066, 047P06Z, 047P076, 047P07Z, 047P0D1, 047P0D6, 047P0DZ, 047P0E6, 047P0EZ, 047P0F6, 047P0FZ, 047P0G6, 047P0GZ, 047P0Z1, 047P0Z6, 047P0ZZ, 047P341, 047P346, 047P34Z, 047P356, 047P35Z, 047P366, 047P36Z, 047P376, 047P37Z, 047P3D1, 047P3D6, 047P3DZ, 047P3E6, 047P3EZ, 047P3F6, 047P3FZ, 047P3G6, 047P3GZ, 047P3Z1, 047P3Z6, 047P3ZZ, 047P441, 047P446, 047P44Z, 047P456, 047P45Z, 047P466, 047P46Z, 047P476, 047P47Z, 047P4D1, 047P4D6, 047P4DZ, 047P4E6, 047P4EZ, 047P4F6, 047P4FZ, 047P4G6, 047P4GZ, 047P4Z1, 047P4Z6, 047P4ZZ, 047Q041, 047Q046, 047Q04Z, 047Q056, 047Q05Z, 047Q066, 047Q06Z, 047Q076, 047Q07Z, 047Q0D1, 047Q0D6, 047Q0DZ, 047Q0E6, 047Q0EZ, 047Q0F6, 047Q0FZ, 047Q0G6, 047Q0GZ, 047Q0Z1, 047Q0Z6, 047Q0ZZ, 047Q341, 047Q346, 047Q34Z, 047Q356, 047Q35Z, 047Q366, 047Q36Z, 047Q376, 047Q37Z, 047Q3D1, 047Q3D6, 047Q3DZ, 047Q3E6, 047Q3EZ, 047Q3F6, 047Q3FZ, 047Q3G6, 047Q3GZ, 047Q3Z1, 047Q3Z6, 047Q3ZZ, 047Q441, 047Q446, 047Q44Z, 047Q456, 047Q45Z, 047Q466, 047Q46Z, 047Q476, 047Q47Z, 047Q4D1, 047Q4D6, 047Q4DZ, 047Q4E6, 047Q4EZ, 047Q4F6, 047Q4FZ, 047Q4G6, 047Q4GZ, 047Q4Z1, 047Q4Z6, 047Q4ZZ, 047R041, 047R046, 047R04Z, 047R056, 047R05Z, 047R066, 047R06Z, 047R076, 047R07Z, 047R0D1, 047R0D6, 047R0DZ, 047R0E6, 047R0EZ, 047R0F6, 047R0FZ, 047R0G6, 047R0GZ, 047R0Z1, 047R0Z6, 047R0ZZ, 047R341, 047R346, 047R34Z, 047R356, 047R35Z, 047R366, 047R36Z, 047R376, 047R37Z, 047R3D1, 047R3D6, 047R3DZ, 047R3E6, 047R3EZ, 047R3F6, 047R3FZ, 047R3G6, 047R3GZ, 047R3Z1, 047R3Z6, 047R3ZZ, 047R441, 047R446, 047R44Z, 047R456, 047R45Z, 047R466, 047R46Z, 047R476, 047R47Z, 047R4D1, 047R4D6, 047R4DZ, 047R4E6, 047R4EZ, 047R4F6, 047R4FZ, 047R4G6, 047R4GZ, 047R4Z1, 047R4Z6, 047R4ZZ, 047S041, 047S046, 047S04Z, 047S056, 047S05Z, 047S066, 047S06Z, 047S076, 047S07Z, 047S0D1, 047S0D6, 047S0DZ, 047S0E6, 047S0EZ, 047S0F6, 047S0FZ, 047S0G6, 047S0GZ, 047S0Z1, 047S0Z6, 047S0ZZ, 047S341, 047S346, 047S34Z, 047S356, 047S35Z, 047S366, 047S36Z, 047S376, 047S37Z, 047S3D1, 047S3D6, 047S3DZ, 047S3E6, 047S3EZ,  047S3F6, 047S3FZ, 047S3G6, 047S3GZ, 047S3Z1, 047S3Z6, 047S3ZZ, 047S441, 047S446, 047S44Z, 047S456, 047S45Z, 047S466, 047S46Z, 047S476, 047S47Z, 047S4D1, 047S4D6, 047S4DZ, 047S4E6, 047S4EZ, 047S4F6, 047S4FZ, 047S4G6, 047S4GZ, 047S4Z1, 047S4Z6, 047S4ZZ, 047T041, 047T046, 047T04Z, 047T056, 047T05Z, 047T066, 047T06Z, 047T076, 047T07Z, 047T0D1, 047T0D6, 047T0DZ, 047T0E6, 047T0EZ, 047T0F6, 047T0FZ, 047T0G6, 047T0GZ, 047T0Z1, 047T0Z6, 047T0ZZ, 047T341, 047T346, 047T34Z, 047T356, 047T35Z, 047T366, 047T36Z, 047T376, 047T37Z, 047T3D1, 047T3D6, 047T3DZ, 047T3E6, 047T3EZ, 047T3F6, 047T3FZ, 047T3G6, 047T3GZ, 047T3Z1, 047T3Z6, 047T3ZZ, 047T441, 047T446, 047T44Z, 047T456, 047T45Z, 047T466, 047T46Z, 047T476, 047T47Z, 047T4D1, 047T4D6, 047T4DZ, 047T4E6, 047T4EZ, 047T4F6, 047T4FZ, 047T4G6, 047T4GZ, 047T4Z1, 047T4Z6, 047T4ZZ, 047U041, 047U046, 047U04Z, 047U056, 047U05Z, 047U066, 047U06Z, 047U076, 047U07Z, 047U0D1, 047U0D6, 047U0DZ, 047U0E6, 047U0EZ, 047U0F6, 047U0FZ, 047U0G6, 047U0GZ, 047U0Z1, 047U0Z6, 047U0ZZ, 047U341, 047U346, 047U34Z, 047U356, 047U35Z, 047U366, 047U36Z, 047U376, 047U37Z, 047U3D1, 047U3D6, 047U3DZ, 047U3E6, 047U3EZ, 047U3F6, 047U3FZ, 047U3G6, 047U3GZ, 047U3Z1, 047U3Z6, 047U3ZZ, 047U441, 047U446, 047U44Z, 047U456, 047U45Z, 047U466, 047U46Z, 047U476, 047U47Z, 047U4D1, 047U4D6, 047U4DZ, 047U4E6, 047U4EZ, 047U4F6, 047U4FZ, 047U4G6, 047U4GZ, 047U4Z1, 047U4Z6, 047U4ZZ, 047V041, 047V046, 047V04Z, 047V056, 047V05Z, 047V066, 047V06Z, 047V076, 047V07Z, 047V0D1, 047V0D6, 047V0DZ, 047V0E6, 047V0EZ, 047V0F6, 047V0FZ, 047V0G6, 047V0GZ, 047V0Z1, 047V0Z6, 047V0ZZ, 047V341, 047V346, 047V34Z, 047V356, 047V35Z, 047V366, 047V36Z, 047V376, 047V37Z, 047V3D1, 047V3D6, 047V3DZ, 047V3E6, 047V3EZ, 047V3F6, 047V3FZ, 047V3G6, 047V3GZ, 047V3Z1, 047V3Z6, 047V3ZZ, 047V441, 047V446, 047V44Z, 047V456, 047V45Z, 047V466, 047V46Z, 047V476, 047V47Z, 047V4D1, 047V4D6, 047V4DZ, 047V4E6, 047V4EZ, 047V4F6, 047V4FZ, 047V4G6, 047V4GZ, 047V4Z1, 047V4Z6, 047V4ZZ, 047W041, 047W046, 047W04Z, 047W056, 047W05Z, 047W066, 047W06Z, 047W076, 047W07Z, 047W0D1, 047W0D6, 047W0DZ, 047W0E6, 047W0EZ, 047W0F6, 047W0FZ, 047W0G6, 047W0GZ, 047W0Z1, 047W0Z6, 047W0ZZ, 047W341, 047W346, 047W34Z, 047W356, 047W35Z, 047W366, 047W36Z, 047W376, 047W37Z, 047W3D1, 047W3D6, 047W3DZ, 047W3E6, 047W3EZ, 047W3F6, 047W3FZ, 047W3G6, 047W3GZ, 047W3Z1, 047W3Z6, 047W3ZZ, 047W441, 047W446, 047W44Z, 047W456, 047W45Z, 047W466, 047W46Z, 047W476, 047W47Z, 047W4D1,  047W4D6, 047W4DZ, 047W4E6, 047W4EZ, 047W4F6, 047W4FZ, 047W4G6, 047W4GZ, 047W4Z1, 047W4Z6, 047W4ZZ, 047Y041, 047Y046, 047Y04Z, 047Y056, 047Y05Z, 047Y066, 047Y06Z, 047Y076, 047Y07Z, 047Y0D1, 047Y0D6, 047Y0DZ, 047Y0E6, 047Y0EZ, 047Y0F6, 047Y0FZ, 047Y0G6, 047Y0GZ, 047Y0Z1, 047Y0Z6, 047Y0ZZ, 047Y341, 047Y346, 047Y34Z, 047Y356, 047Y35Z, 047Y366, 047Y36Z, 047Y376, 047Y37Z, 047Y3D1, 047Y3D6, 047Y3DZ, 047Y3E6, 047Y3EZ, 047Y3F6, 047Y3FZ, 047Y3G6, 047Y3GZ, 047Y3Z1, 047Y3Z6, 047Y3ZZ, 047Y441, 047Y446, 047Y44Z, 047Y456, 047Y45Z, 047Y466, 047Y46Z, 047Y476, 047Y47Z, 047Y4D1, 047Y4D6, 047Y4DZ, 047Y4E6, 047Y4EZ, 047Y4F6, 047Y4FZ, 047Y4G6, 047Y4GZ, 047Y4Z1, 047Y4Z6, 047Y4ZZ, 04CC0Z6, 04CC0ZZ, 04CC3Z6, 04CC3ZZ, 04CC4Z6, 04CC4ZZ, 04CD0Z6, 04CD0ZZ, 04CD3Z6, 04CD3ZZ, 04CD4Z6, 04CD4ZZ, 04CE0Z6, 04CE0ZZ, 04CE3Z6, 04CE3ZZ, 04CE4Z6, 04CE4ZZ, 04CF0Z6, 04CF0ZZ, 04CF3Z6, 04CF3ZZ, 04CF4Z6, 04CF4ZZ, 04CH0Z6, 04CH0ZZ, 04CH3Z6, 04CH3ZZ, 04CH4Z6, 04CH4ZZ, 04CJ0Z6, 04CJ0ZZ, 04CJ3Z6, 04CJ3ZZ, 04CJ4Z6, 04CJ4ZZ, 04CK0Z6, 04CK0ZZ, 04CK3Z6, 04CK3ZZ, 04CK4Z6, 04CK4ZZ, 04CL0Z6, 04CL0ZZ, 04CL3Z6, 04CL3ZZ, 04CL4Z6, 04CL4ZZ, 04CM0Z6, 04CM0ZZ, 04CM3Z6, 04CM3ZZ, 04CM4Z6, 04CM4ZZ, 04CN0Z6, 04CN0ZZ, 04CN3Z6, 04CN3ZZ, 04CN4Z6, 04CN4ZZ, 04CP0Z6, 04CP0ZZ, 04CP3Z6, 04CP3ZZ, 04CP4Z6, 04CP4ZZ, 04CQ0Z6, 04CQ0ZZ, 04CQ3Z6, 04CQ3ZZ, 04CQ4Z6, 04CQ4ZZ, 04CR0Z6, 04CR0ZZ, 04CR3Z6, 04CR3ZZ, 04CR4Z6, 04CR4ZZ, 04CS0Z6, 04CS0ZZ, 04CS3Z6, 04CS3ZZ, 04CS4Z6, 04CS4ZZ, 04CT0Z6, 04CT0ZZ, 04CT3Z6, 04CT3ZZ, 04CT4Z6, 04CT4ZZ, 04LE4CT, 04CU0Z6, 04CU0ZZ, 04CU3Z6, 04CU3ZZ, 04CU4Z6, 04CU4ZZ, 04CV0Z6, 04CV0ZZ, 04CV3Z6, 04CV3ZZ, 04CV4Z6, 04CV4ZZ, 04CW0Z6, 04CW0ZZ, 04CW3Z6, 04CW3ZZ, 04CW4Z6, 04CW4ZZ, 04CY0Z6, 04CY0ZZ, 04CY3Z6, 04CY3ZZ, 04CY4Z6, 04CY4ZZ |
|  | CPT: 37220, 37221, 37222, 37223, 37224, 37225, 37226, 37227, 37228, 37229, 37230, 37231, 37232, 37233, 37234, 37235 |
| Surgical revascularization | ICD-9: 3925, 3929, 3808, 3816, 3818, 3838, 3848, 3868, 3888 |
|  | ICD-10: 0410096, 0410097, 0410098, 0410099, 041009B, 041009C, 041009D, 041009F, 041009G, 041009H, 041009J, 041009K, 041009Q, 041009R, 04100A6, 04100A7, 04100A8, 04100A9, 04100AB, 04100AC, 04100AD, 04100AF, 04100AG, 04100AH, 04100AJ, 04100AK, 04100AQ, 04100AR, 04100J6, 04100J7, 04100J8, 04100J9, 04100JB, 04100JC, 04100JD, 04100JF, 04100JG, 04100JH, 04100JJ, 04100JK, 04100JQ, 04100JR, 04100K6, 04100K7, 04100K8, 04100K9, 04100KB, 04100KC, 04100KD, 04100KF, 04100KG, 04100KH, 04100KJ, 04100KK, 04100KQ, 04100KR, 04100Z6, 04100Z7, 04100Z8, 04100Z9,  04100ZB, 04100ZC, 04100ZD, 04100ZF, 04100ZG, 04100ZH, 04100ZJ, 04100ZK, 04100ZQ, 04100ZR, 0410496, 0410497, 0410498, 0410499, 041049B, 041049C, 041049D, 041049F, 041049G, 041049H, 041049J, 041049K, 041049Q, 041049R, 04104A6, 04104A7, 04104A8, 04104A9, 04104AB, 04104AC, 04104AD, 04104AF, 04104AG, 04104AH, 04104AJ, 04104AK, 04104AQ, 04104AR, 04104J6, 04104J7, 04104J8, 04104J9, 04104JB, 04104JC, 04104JD, 04104JF, 04104JG, 04104JH, 04104JJ, 04104JK, 04104JQ, 04104JR, 04104K6, 04104K7, 04104K8, 04104K9, 04104KB, 04104KC, 04104KD, 04104KF, 04104KG, 04104KH, 04104KJ, 04104KK, 04104KQ, 04104KR, 04104Z6, 04104Z7, 04104Z8, 04104Z9, 04104ZB, 04104ZC, 04104ZD, 04104ZF, 04104ZG, 04104ZH, 04104ZJ, 04104ZK, 04104ZQ, 04104ZR, 041C09H, 041C09J, 041C09K, 041C0AH, 041C0AJ, 041C0AK, 041C0JH, 041C0JJ, 041C0JK, 041C0KH, 041C0KJ, 041C0KK, 041C0ZH, 041C0ZJ, 041C0ZK, 041C49H, 041C49J, 041C49K, 041C4AH, 041C4AJ, 041C4AK, 041C4JH, 041C4JJ, 041C4JK, 041C4KH, 041C4KJ, 041C4KK, 041C4ZH, 041C4ZJ, 041C4ZK, 041D09H, 041D09J, 041D09K, 041D0AH, 041D0AJ, 041D0AK, 041D0JH, 041D0JJ, 041D0JK, 041D0KH, 041D0KJ, 041D0KK, 041D0ZH, 041D0ZJ, 041D0ZK, 041D49H, 041D49J, 041D49K, 041D4AH, 041D4AJ, 041D4AK, 041D4JH, 041D4JJ, 041D4JK, 041D4KH, 041D4KJ, 041D4KK, 041D4ZH, 041D4ZJ, 041D4ZK, 041E09H, 041E09J, 041E09K, 041E09P, 041E09Q, 041E0AH, 041E0AJ, 041E0AK, 041E0AP, 041E0AQ, 041E0JH, 041E0JJ, 041E0JK, 041E0JP, 041E0JQ, 041E0KH, 041E0KJ, 041E0KK, 041E0KP, 041E0KQ, 041E0ZH, 041E0ZJ, 041E0ZK, 041E0ZP, 041E0ZQ, 041E49H, 041E49J, 041E49K, 041E49P, 041E49Q, 041E4JH, 041E4JJ, 041E4JK, 041E4JP, 041E4JQ, 041E4KH, 041E4KJ, 041E4KK, 041E4KP, 041E4KQ, 041E4ZH, 041E4ZJ, 041E4ZK, 041E4ZP, 041E4ZQ, 041F09H, 041F09J, 041F09K, 041F09P, 041F09Q, 041F0AH, 041F0AJ, 041F0AK, 041F0AP, 041F0AQ, 041F0JH, 041F0JJ, 041F0JK, 041F0JP, 041F0JQ, 041F0KH, 041F0KJ, 041F0KK, 041F0KP, 041F0KQ, 041F0ZH, 041F0ZJ, 041F0ZK, 041F0ZP, 041F0ZQ, 041F49H, 041F49J, 041F49K, 041F49P, 041F49Q, 041F4AH, 041F4AJ, 041F4AK, 041F4AP, 041F4AQ, 041F4JH, 041F4JJ, 041F4JK, 041F4JP, 041F4JQ, 041F4KH, 041F4KJ, 041F4KK, 041F4KP, 041F4KQ, 041F4ZH, 041F4ZJ, 041F4ZK, 041F4ZP, 041F4ZQ, 041H09H, 041H09J, 041H09K, 041H09P, 041H09Q, 041H0AH, 041H0AJ, 041H0AK, 041H0AP, 041H0AQ, 041H0JH, 041H0JJ, 041H0JK, 041H0JP, 041H0JQ, 041H0KH, 041H0KJ, 041H0KK, 041H0KP, 041H0KQ, 041H0ZH, 041H0ZJ, 041H0ZK, 041H0ZP, 041H0ZQ, 041H49H, 041H49J, 041H49K, 041H49P, 041H49Q, 041H4AH, 041H4AJ, 041H4AK, 041H4AP, 041H4AQ, 041H4JH, 041H4JJ, 041H4JK, 041H4JP, 041H4JQ, 041H4KH,  041H4KJ, 041H4KK, 041H4KP, 041H4KQ, 041H4ZH, 041H4ZJ, 041H4ZK, 041H4ZP, 041H4ZQ, 041J09H, 041J09J, 041J09K, 041J09P, 041J09Q, 041J0AH, 041J0AJ, 041J0AK, 041J0AP, 041J0AQ, 041J0JH, 041J0JJ, 041J0JK, 041J0JP, 041J0JQ, 041J0KH, 041J0KJ, 041J0KK, 041J0KP, 041J0KQ, 041J0ZH, 041J0ZJ, 041J0ZK, 041J0ZP, 041J0ZQ, 041J49H, 041J49J, 041J49K, 041J49P, 041J49Q, 041J4AH, 041J4AJ, 041J4AK, 041J4AP, 041J4AQ, 041J4JH, 041J4JJ, 041J4JK, 041J4JP, 041J4JQ, 041J4KH, 041J4KJ, 041J4KK, 041J4KP, 041J4KQ, 041J4ZH, 041J4ZJ, 041J4ZK, 041J4ZP, 041J4ZQ, 0410090, 0410091, 0410092, 04100A0, 041C096, 041C097, 041C098, 041C099, 041C09B, 041C09C, 041C09D, 041C09F, 041C09G, 041C09H, 041C09J, 041C09K, 041C09Q, 041C09R, 041C0A6, 041C0A7, 041C0A8, 041C0A9, 041C0AB, 041C0AC, 041C0AD, 041C0AF, 041C0AG, 041C0AH, 041C0AJ, 041C0AK, 041C0AQ, 041C0AR, 041C0J6, 041C0J7, 041C0J8, 041C0J9, 041C0JB, 041C0JC, 041C0JD, 041C0JF, 041C0JG, 041C0JH, 041C0JJ, 041C0JK, 041C0JQ, 041C0JR, 041C0K6, 041C0K7, 041C0K8, 041C0K9, 041C0KB, 041C0KC, 041C0KD, 041C0KF, 041C0KG, 041C0KH, 041C0KJ, 041C0KK, 041C0KQ, 041C0KR, 041C0Z6, 041C0Z7, 041C0Z8, 041C0Z9, 041C0ZB, 041C0ZC, 041C0ZD, 041C0ZF, 041C0ZG, 041C0ZH, 041C0ZJ, 041C0ZK, 041C0ZQ, 041C0ZR, 041C496, 041C497, 041C498, 041C499, 041C49B, 041C49C, 041C49D, 041C49F, 041C49G, 041C49H, 041C49J, 041C49K, 041C49Q, 041C49R, 041C4A6, 041C4A7, 041C4A8, 041C4A9, 041C4AB, 041C4AC, 041C4AD, 041C4AF, 041C4AG, 041C4AH, 041C4AJ, 041C4AK, 041C4AQ, 041C4AR, 041C4J6, 041C4J7, 041C4J8, 041C4J9, 041C4JB, 041C4JC, 041C4JD, 041C4JF, 041C4JG, 041C4JH, 041C4JJ, 041C4JK, 041C4JQ, 041C4JR, 041C4K6, 041C4K7, 041C4K8, 041C4K9, 041C4KB, 041C4KC, 041C4KD, 041C4KF, 041C4KG, 041C4KH, 041C4KJ, 041C4KK, 041C4KQ, 041C4KR, 041C4Z6, 041C4Z7, 041C4Z8, 041C4Z9, 041C4ZB, 041C4ZC, 041C4ZD, 041C4ZF, 041C4ZG, 041C4ZH, 041C4ZJ, 041C4ZK, 041C4ZQ, 041C4ZR, 041D096, 041D097, 041D098, 041D099, 041D09B, 041D09C, 041D09D, 041D09F, 041D09G, 041D09H, 041D09J, 041D09K, 041D09Q, 041D09R, 041D0A6, 041D0A7, 041D0A8, 041D0A9, 041D0AB, 041D0AC, 041D0AD, 041D0AF, 041D0AG, 041D0AH, 041D0AJ, 041D0AK, 041D0AQ, 041D0AR, 041D0J6, 041D0J7, 041D0J8, 041D0J9, 041D0JB, 041D0JC, 041D0JD, 041D0JF, 041D0JG, 041D0JH, 041D0JJ, 041D0JK, 041D0JQ, 041D0JR, 041D0K6, 041D0K7, 041D0K8, 041D0K9, 041D0KB, 041D0KC, 041D0KD, 041D0KF, 041D0KG, 041D0KH, 041D0KJ, 041D0KK, 041D0KQ, 041D0KR, 041D0Z6, 041D0Z7, 041D0Z8, 041D0Z9, 041D0ZB,  041D0ZC, 041D0ZD, 041D0ZF, 041D0ZG, 041D0ZH, 041D0ZJ, 041D0ZK, 041D0ZQ, 041D0ZR, 041D490, 041D496, 041D497, 041D498, 041D499, 041D49B, 041D49C, 041D49D, 041D49F, 041D49G, 041D49H, 041D49J, 041D49K, 041D49Q, 041D49R, 041D4A0, 041D4A6, 041D4A7, 041D4A8, 041D4A9, 041D4AB, 041D4AC, 041D4AD, 041D4AF, 041D4AG, 041D4AH, 041D4AJ, 041D4AK, 041D4AQ, 041D4AR, 041D4J0, 041D4J6, 041D4J7, 041D4J8, 041D4J9, 041D4JB, 041D4JC, 041D4JD, 041D4JF, 041D4JG, 041D4JH, 041D4JJ, 041D4JK, 041D4JQ, 041D4JR, 041D4K0, 041D4K6, 041D4K7, 041D4K8, 041D4K9, 041D4KB, 041D4KC, 041D4KD, 041D4KF, 041D4KG, 041D4KH, 041D4KJ, 041D4KK, 041D4KQ, 041D4KR, 041D4Z0, 041D4Z6, 041D4Z7, 041D4Z8, 041D4Z9, 041D4ZB, 041D4ZC, 041D4ZD, 041D4ZF, 041D4ZG, 041D4ZH, 041D4ZJ, 041D4ZK, 041D4ZQ, 041D4ZR, 041E099, 041E09B, 041E09C, 041E09D, 041E09F, 041E09G, 041E09H, 041E09J, 041E09K, 041E09P, 041E09Q, 041E0A9, 041E0AB, 041E0AC, 041E0AD, 041E0AF, 041E0AG, 041E0AH, 041E0AJ, 041E0AK, 041E0AP, 041E0AQ, 041E0J9, 041E0JB, 041E0JC, 041E0JD, 041E0JF, 041E0JG, 041E0JH, 041E0JJ, 041E0JK, 041E0JP, 041E0JQ, 041E0K9, 041E0KB, 041E0KC, 041E0KD, 041E0KF, 041E0KG, 041E0KH, 041E0KJ, 041E0KK, 041E0KP, 041E0KQ, 041E0Z9, 041E0ZB, 041E0ZC, 041E0ZD, 041E0ZF, 041E0ZG, 041E0ZH, 041E0ZJ, 041E0ZK, 041E0ZP, 041E0ZQ, 041E499, 041E49B, 041E49C, 041E49D, 041E49F, 041E49G, 041E49H, 041E49J, 041E49K, 041E49P, 041E49Q, 041E4A9, 041E4AB, 041E4AC, 041E4AD, 041E4AF, 041E4AG, 041E4AH, 041E4AJ, 041E4AK, 041E4AP, 041E4AQ, 041E4J9, 041E4JB, 041E4JC, 041E4JD, 041E4JF, 041E4JG, 041E4JH, 041E4JJ, 041E4JK, 041E4JP, 041E4JQ, 041E4K9, 041E4KB, 041E4KC, 041E4KD, 041E4KF, 041E4KG, 041E4KH, 041E4KJ, 041E4KK, 041E4KP, 041E4KQ, 041E4Z9, 041E4ZB, 041E4ZC, 041E4ZD, 041E4ZF, 041E4ZG, 041E4ZH, 041E4ZJ, 041E4ZK, 041E4ZP, 041E4ZQ, 041F099, 041F09B, 041F09C, 041F09D, 041F09F, 041F09G, 041F09H, 041F09J, 041F09K, 041F09P, 041F09Q, 041F0A9, 041F0AB, 041F0AC, 041F0AD, 041F0AF, 041F0AG, 041F0AH, 041F0AJ, 041F0AK, 041F0AP, 041F0AQ, 041F0J9, 041F0JB, 041F0JC, 041F0JD, 041F0JF, 041F0JG, 041F0JH, 041F0JJ, 041F0JK, 041F0JP, 041F0JQ, 041F0K9, 041F0KB, 041F0KC, 041F0KD, 041F0KF, 041F0KG, 041F0KH, 041F0KJ, 041F0KK, 041F0KP, 041F0KQ, 041F0Z9, 041F0ZB, 041F0ZC, 041F0ZD, 041F0ZF, 041F0ZG, 041F0ZH, 041F0ZJ, 041F0ZK, 041F0ZP, 041F0ZQ, 041F499, 041F49B, 041F49C, 041F49D, 041F49F, 041F49G, 041F49H, 041F49J, 041F49K, 041F49P, 041F49Q, 041F4A9, 041F4AB, 041F4AC, 041F4AD, 041F4AF, 041F4AG, 041F4AH, 041F4AJ, 041F4AK,  041F4AP, 041F4AQ, 041F4J9, 041F4JB, 041F4JC, 041F4JD, 041F4JF, 041F4JG, 041F4JH, 041F4JJ, 041F4JK, 041F4JP, 041F4JQ, 041F4K9, 041F4KB, 041F4KC, 041F4KD, 041F4KF, 041F4KG, 041F4KH, 041F4KJ, 041F4KK, 041F4KP, 041F4KQ, 041F4Z9, 041F4ZB, 041F4ZC, 041F4ZD, 041F4ZF, 041F4ZG, 041F4ZH, 041F4ZJ, 041F4ZK, 041F4ZP, 041F4ZQ, 041H099, 041H09B, 041H09C, 041H09D, 041H09F, 041H09G, 041H09H, 041H09J, 041H09K, 041H09P, 041H09Q, 041H0A9, 041H0AB, 041H0AC, 041H0AD, 041H0AF, 041H0AG, 041H0AH, 041H0AJ, 041H0AK, 041H0AP, 041H0AQ, 041H0J9, 041H0JB, 041H0JC, 041H0JD, 041H0JF, 041H0JG, 041H0JH, 041H0JJ, 041H0JK, 041H0JP, 041H0JQ, 041H0K9, 041H0KB, 041H0KC, 041H0KD, 041H0KF, 041H0KG, 041H0KH, 041H0KJ, 041H0KK, 041H0KP, 041H0KQ, 041H0Z9, 041H0ZB, 041H0ZC, 041H0ZD, 041H0ZF, 041H0ZG, 041H0ZH, 041H0ZJ, 041H0ZK, 041H0ZP, 041H0ZQ, 041H499, 041H49B, 041H49C, 041H49D, 041H49F, 041H49G, 041H49H, 041H49J, 041H49K, 041H49P, 041H49Q, 041H4A9, 041H4AB, 041H4AC, 041H4AD, 041H4AF, 041H4AG, 041H4AH, 041H4AJ, 041H4AK, 041H4AP, 041H4AQ, 041H4J9, 041H4JB, 041H4JC, 041H4JD, 041H4JF, 041H4JG, 041H4JH, 041H4JJ, 041H4JK, 041H4JP, 041H4JQ, 041H4K9, 041H4KB, 041H4KC, 041H4KD, 041H4KF, 041H4KG, 041H4KH, 041H4KJ, 041H4KK, 041H4KP, 041H4KQ, 041H4Z9, 041H4ZB, 041H4ZC, 041H4ZD, 041H4ZF, 041H4ZG, 041H4ZH, 041H4ZJ, 041H4ZK, 041H4ZP, 041H4ZQ, 041J099, 041J09B, 041J09C, 041J09D, 041J09F, 041J09G, 041J09H, 041J09J, 041J09K, 041J09P, 041J09Q, 041J0A9, 041J0AB, 041J0AC, 041J0AD, 041J0AF, 041J0AG, 041J0AH, 041J0AJ, 041J0AK, 041J0AP, 041J0AQ, 041J0J9, 041J0JB, 041J0JC, 041J0JD, 041J0JF, 041J0JG, 041J0JH, 041J0JJ, 041J0JK, 041J0JP, 041J0JQ, 041J0K9, 041J0KB, 041J0KC, 041J0KD, 041J0KF, 041J0KG, 041J0KH, 041J0KJ, 041J0KK, 041J0KP, 041J0KQ, 041J0Z9, 041J0ZB, 041J0ZC, 041J0ZD, 041J0ZF, 041J0ZG, 041J0ZH, 041J0ZJ, 041J0ZK, 041J0ZP, 041J0ZQ, 041J499, 041J49B, 041J49C, 041J49D, 041J49F, 041J49G, 041J49H, 041J49J, 041J49K, 041J49P, 041J49Q, 041J4A9, 041J4AB, 041J4AC, 041J4AD, 041J4AF, 041J4AG, 041J4AH, 041J4AJ, 041J4AK, 041J4AP, 041J4AQ, 041J4J9, 041J4JB, 041J4JC, 041J4JD, 041J4JF, 041J4JG, 041J4JH, 041J4JJ, 041J4JK, 041J4JP, 041J4JQ, 041J4K9, 041J4KB, 041J4KC, 041J4KD, 041J4KF, 041J4KG, 041J4KH, 041J4KJ, 041J4KK, 041J4KP, 041J4KQ, 041J4Z9, 041J4ZB, 041J4ZC, 041J4ZD, 041J4ZF, 041J4ZG, 041J4ZH, 041J4ZJ, 041J4ZK, 041J4ZP, 041J4ZQ, 0312096, 0312097, 0312098, 0312099, 031209B, 031209C, 03120A6, 03120A7, 03120A8, 03120A9, 03120AB, 03120AC, 03120J6, 03120J7,  03120J8, 03120J9, 03120JB, 03120JC, 03120K6, 03120K7, 03120K8, 03120K9, 03120KB, 03120KC, 03120Z6, 03120Z7, 03120Z8, 03120Z9, 03120ZB, 03120ZC, 0313096, 0313097, 0313098, 0313099, 031309B, 031309C, 03130A6, 03130A7, 03130A8, 03130A9, 03130AB, 03130AC, 03130J6, 03130J7, 03130J8, 03130J9, 03130JB, 03130JC, 03130K6, 03130K7, 03130K8, 03130K9, 03130KB, 03130KC, 03130Z6, 03130Z7, 03130Z8, 03130Z9, 03130ZB, 03130ZC, 0314096, 0314097, 0314098, 0314099, 031409B, 031409C, 03140A6, 03140A7, 03140A8, 03140A9, 03140AB, 03140AC, 03140J6, 03140J7, 03140J8, 03140J9, 03140JB, 03140JC, 03140K6, 03140K7, 03140K8, 03140K9, 03140KB, 03140KC, 03140Z6, 03140Z7, 03140Z8, 03140Z9, 03140ZB, 03140ZC, 0315096, 0315097, 0315098, 0315099, 031509B, 031509C, 03150A6, 03150A7, 03150A8, 03150A9, 03150AB, 03150AC, 03150J6, 03150J7, 03150J8, 03150J9, 03150JB, 03150JC, 03150K6, 03150K7, 03150K8, 03150K9, 03150KB, 03150KC, 03150Z6, 03150Z7, 03150Z8, 03150Z9, 03150ZB, 03150ZC, 0316096, 0316097, 0316098, 0316099, 031609B, 031609C, 03160A6, 03160A7, 03160A8, 03160A9, 03160AB, 03160AC, 03160J6, 03160J7, 03160J8, 03160J9, 03160JB, 03160JC, 03160K6, 03160K7, 03160K8, 03160K9, 03160KB, 03160KC, 03160Z6, 03160Z7, 03160Z8, 03160Z9, 03160ZB, 03160ZC, 041K09H, 041K09J, 041K09K, 041K09L, 041K09M, 041K09N, 041K09P, 041K09Q, 041K09S, 041K0AH, 041K0AJ, 041K0AK, 041K0AL, 041K0AM, 041K0AN, 041K0AP, 041K0AQ, 041K0AS, 041K0JH, 041K0JJ, 041K0JK, 041K0JL, 041K0JM, 041K0JN, 041K0JP, 041K0JQ, 041K0JS, 041K0KH, 041K0KJ, 041K0KK, 041K0KL, 041K0KM, 041K0KN, 041K0KP, 041K0KQ, 041K0KS, 041K0ZH, 041K0ZJ, 041K0ZK, 041K0ZL, 041K0ZM, 041K0ZN, 041K0ZP, 041K0ZQ, 041K0ZS, 041K3JQ, 041K3JS, 041K49H, 041K49J, 041K49K, 041K49L, 041K49M, 041K49N, 041K49P, 041K49Q, 041K49S, 041K4AH, 041K4AJ, 041K4AK, 041K4AL, 041K4AM, 041K4AN, 041K4AP, 041K4AQ, 041K4AS, 041K4JH, 041K4JJ, 041K4JK, 041K4JL, 041K4JM, 041K4JN, 041K4JP, 041K4JQ, 041K4JS, 041K4KH, 041K4KJ, 041K4KK, 041K4KL, 041K4KM, 041K4KN, 041K4KP, 041K4KQ, 041K4KS, 041K4ZH, 041K4ZJ, 041K4ZK, 041K4ZL, 041K4ZM, 041K4ZN, 041K4ZP, 041K4ZQ, 041K4ZS, 041L09H, 041L09J, 041L09K, 041L09L, 041L09M, 041L09N, 041L09P, 041L09Q, 041L0AH, 041L0AJ, 041L0AK, 041L0AL, 041L0AM, 041L0AN, 041L0AP, 041L0AQ, 041L0JH, 041L0JJ, 041L0JK, 041L0JL, 041L0JM, 041L0JN, 041L0JP, 041L0JQ, 041L0KH, 041L0KJ, 041L0KK, 041L0KL, 041L0KM, 041L0KN, 041L0KP, 041L0KQ, 041L0ZH, 041L0ZJ, 041L0ZK, 041L0ZL, 041L0ZM, 041L0ZN, 041L0ZP, 041L0ZQ, 041L3JQ, 041L3JS, 041L49H, 041L49J, 041L49K, 041L49L, 041L49M, 041L49N, 041L49P, 041L49Q, 041L4AH, 041L4AJ,  041L4AK, 041L4AL, 041L4AM, 041L4AN, 041L4AP, 041L4AQ, 041L4JH, 041L4JJ, 041L4JK, 041L4JL, 041L4JM, 041L4JN, 041L4JP, 041L4JQ, 041L4KH, 041L4KJ, 041L4KK, 041L4KL, 041L4KM, 041L4KN, 041L4KP, 041L4KQ, 041L4ZH, 041L4ZJ, 041L4ZK, 041L4ZL, 041L4ZM, 041L4ZN, 041L4ZP, 041L4ZQ, 041M09L, 041M09M, 041M09P, 041M09Q, 041M0AL, 041M0AM, 041M0AP, 041M0AQ, 041M0JL, 041M0JM, 041M0JP, 041M0JQ, 041M0KL, 041M0KM, 041M0KP, 041M0KQ, 041M0ZL, 041M0ZM, 041M0ZP, 041M0ZQ, 041M3JQ, 041M49L, 041M49M, 041M49P, 041M49Q, 041M4AL, 041M4AM, 041M4AP, 041M4AQ, 041M4JL, 041M4JM, 041M4JP, 041M4JQ, 041M4KL, 041M4KM, 041M4KP, 041M4KQ, 041M4ZL, 041M4ZM, 041M4ZP, 041M4ZQ, 041N09L, 041N09M, 041N09P, 041N09Q, 041N0AL, 041N0AM, 041N0AP, 041N0AQ, 041N0JL, 041N0JM, 041N0JP, 041N0JQ, 041N0KL, 041N0KM, 041N0KP, 041N0KQ, 041N0ZL, 041N0ZM, 041N0ZP, 041N0ZQ, 041N3JQ, 041N49L, 041N49M, 041N49P, 041N49Q, 041N4AL, 041N4AM, 041N4AP, 041N4AQ, 041N4JL, 041N4JM, 041N4JP, 041N4JQ, 041N4KL, 041N4KM, 041N4KP, 041N4KQ, 041N4ZL, 041N4ZM, 041N4ZP, 041N4ZQ, 041T09P, 041T09Q, 041T0AP, 041T0AQ, 041T0JP, 041T0JQ, 041T0KP, 041T0KQ, 041T0ZP, 041T0ZQ, 041T3JQ, 041T49P, 041T49Q, 041T4AP, 041T4AQ, 041T4JP, 041T4JQ, 041T4KP, 041T4KQ, 041T4ZP, 041T4ZQ, 041U09P, 041U09Q, 041U0AP, 041U0AQ, 041U0JP, 041U0JQ, 041U0KP, 041U0KQ, 041U0ZP, 041U0ZQ, 041U3JQ, 041U49P, 041U49Q, 041U4AP, 041U4AQ, 041U4JP, 041U4JQ, 041U4KP, 041U4KQ, 041U4ZP, 041U4ZQ, 041V09P, 041V09Q, 041V0AP, 041V0AQ, 041V0JP, 041V0JQ, 041V0KP, 041V0KQ, 041V0ZP, 041V0ZQ, 041V3JQ, 041V49P, 041V49Q, 041V4AP, 041V4AQ, 041V4JP, 041V4JQ, 041V4KP, 041V4KQ, 041V4ZP, 041V4ZQ, 041W09P, 041W09Q, 041W0AP, 041W0AQ, 041W0JP, 041W0JQ, 041W0KP, 041W0KQ, 041W0ZP, 041W0ZQ, 041W3JQ, 041W49P, 041W49Q, 041W4AP, 041W4AQ, 041W4JP, 041W4JQ, 041W4KP, 041W4KQ, 041W4ZP, 041W4ZQ |
|  | CPT: 35621, 35623, 35637, 35638, 35646, 35647, 35654, 35656, 35661, 35663, 35665, 35666, 35671, 35521, 35533, 35556, 35558, 35566, 35570, 35571, 35583, 35585, 35587, 35686 |

**Supplemental Table 2:** Baseline characteristics of patients excluded for lack of lower extremity arterial testing

| **Variable** | **Total (n = 869,859)** |
| --- | --- |
| Age | 76.4 ± 6.0 |
| Female sex | 452,146 (52.0) |
| Race/ethnicity |  |
| White | 720,979 (82.9) |
| Black | 99,677(11.5) |
| Asian | 10,559 (1.2) |
| Hispanic | 18,795 (2.2) |
| Native American | 4,619 (0.5) |
| Other/unknown | 15,230 (1.8) |
| Region |  |
| Midwest | 187,923 (21.6) |
| Northeast | 234,795 (27.0) |
| South | 302,910 (34.8) |
| West | 140,994 (16.2) |
| Rutherford class |  |
| 4 | 404,211 (46.5) |
| 5 | 395,028 (45.4) |
| 6 | 70,620 (8.1) |
| Elixhauser comorbidities | 5.3 ± 3.2 |
| Congestive heart failure | 217,996 (25.1) |
| Valvular heart disease | 128,190 (14.7) |
| Diabetes mellitus | 396,037 (45.5) |
| Hypertension | 656,648 (75.5) |
| Chronic kidney disease | 208,572 (24.0) |
| Chronic lung disease | 227,550 (26.2) |
| Cardiac arrhythmia | 59,023 (6.8) |
| Obesity | 131,929 (15.2) |
| Depression | 143,059 (16.4) |

**Supplemental Table 2:** Proportion of patients with each qualifying diagnosis code or combination of diagnostic codes

| **ICD-9 Diagnosis code(s)** | | **N (%)** |
| --- | --- | --- |
| (250.7x, 249.70, OR 249.71) + 707.1 | diabetes w/ circulatory disorder + ulcer | 57,784 (7.2%) |
| (250.7x, 249.70, OR 249.71) + (730.0x, 730.1x, OR 730.2x) | diabetes w/circulatory disorder + osteomyelitis | 8,915 (1.1%) |
| (250.7x, 249.70, OR 249.71) + 785.4 | diabetes w/ circulatory disorder + gangrene | 9,734 (1.2%) |
| (250.7x, 249.70, OR 249.71) + (682.6, 682.7, OR 681.1) | diabetes w/ circulatory disorder + cellulitis | 31,727 (3.9%) |
| (440.20, 440.21, 440.29, 443.9, OR 440.9) + 707.1 | lower extremity atherosclerosis + ulcer | 127,656 (15.8%) |
| (440.20, 440.21, 440.29, 443.9, OR 440.9) + (730.0x, 730.1x, OR 730.2x) | lower extremity atherosclerosis + osteomyelitis | 14.103 (1.7%) |
| (440.20, 440.21, 440.29, 443.9, OR 440.9) + 785.4 | lower extremity atherosclerosis + gangrene | 11,623 (1.4%) |
| (440.20, 440.21, 440.29, 443.9, OR 440.9) + (682.6, 682.7, OR 681.1) | Lower extremity atherosclerosis + cellulitis | 92,110 (11.4%) |
| 440.22 | lower extremity atherosclerosis w/ rest pain | 292,589 (36.2%) |
| 440.23 | lower extremity atherosclerosis w/ ulcer | 123,941 (15.4%) |
| 440.24 | lower extremity atherosclerosis w/ gangrene | 37,035 (4.6%) |
| **ICD-10 diagnosis code(s)** | | **N (%)** |
| (I70.21x, I70.29x, OR I70.20x) + (M86.1x, M86.2x,M86.3x, M86.4x, M86.6x, M86.7x, M86.8x, OR M86.9) | lower extremity atherosclerosis + osteomyelitis | 2,504 (0.5%) |
| (I70.21x, I70.29x, OR I70.20x) + I96 | lower extremity atherosclerosis + gangrene | 1,312 (0.3%) |
| (I70.21x, I70.29x, OR I70.20x) + (L97.1x, L97.2x L97.3x, L97.4x, L97.5x, L97.6x, L97.8x, OR L97.9x) | lower extremity atherosclerosis + ulcer | 21,093 (4.6%) |
| (I70.21x, I70.29x, OR I70.20x) + (E08.621, E08.622, E09.621, E10.621, E11.621, E13.621, E09.622, E10.622, E11.622, OR E13.622) | lower extremity atherosclerosis + diabetes w/ ulcer | 5,593 (1.2%) |
| (I70.21x, I70.29x, OR I70.20x) + (L03.03x, L03.115, L03.116, OR L03.119) | lower extremity atherosclerosis + cellulitis | 12,942 (2.8%) |
| I73.9 + (M86.1x, M86.2x,M86.3x, M86.4x, M86.6x, M86.7x, M86.8x, OR M86.9) | peripheral vascular disease + osteomyelitis | 8,499 (1.8%) |
| I73.9 + I96 | peripheral vascular disease + gangrene | 5,064 (1.1%) |
| I73.9 + (L97.1x, L97.2x L97.3x, L97.4x, L97.5x, L97.6x, L97.8x, OR L97.9x) | peripheral vascular disease + cellulitis | 60,349 (13.1%) |
| I73.9 + (E08.621, E08.622, E09.621, E10.621, E11.621, E13.621, E09.622, E10.622, E11.622, OR E13.622) | peripheral vascular disease + ulcer | 16,967 (3.7%) |
| I73.9 + (L03.03x, L03.115, L03.116, OR L03.119) | peripheral vascular disease + diabetes w/ ulcer | 43,293 (9.4%) |
| I70.22x | lower extremity atherosclerosis w/ rest pain | 159,647 (34.7%) |
| I70.23x, I70.24x, OR I70.25x | lower extremity atherosclerosis w/ ulcer | 77,418 (16.8%) |
| I70.26x | lower extremity atherosclerosis w/ gangrene | 18,700 (4.1%) |
| E08.52, E09.52, E10.52, E11.52, OR E13.52 | diabetes w/ angiopathy and ulcer | 26,661 (5.8%) |

**Supplemental Figure 1**: Cumulative incidence of all-cause death (A), major amputation (B), and revascularization (C) overall and by Rutherford class among patients excluded for lack of lower extremity arterial testing

**
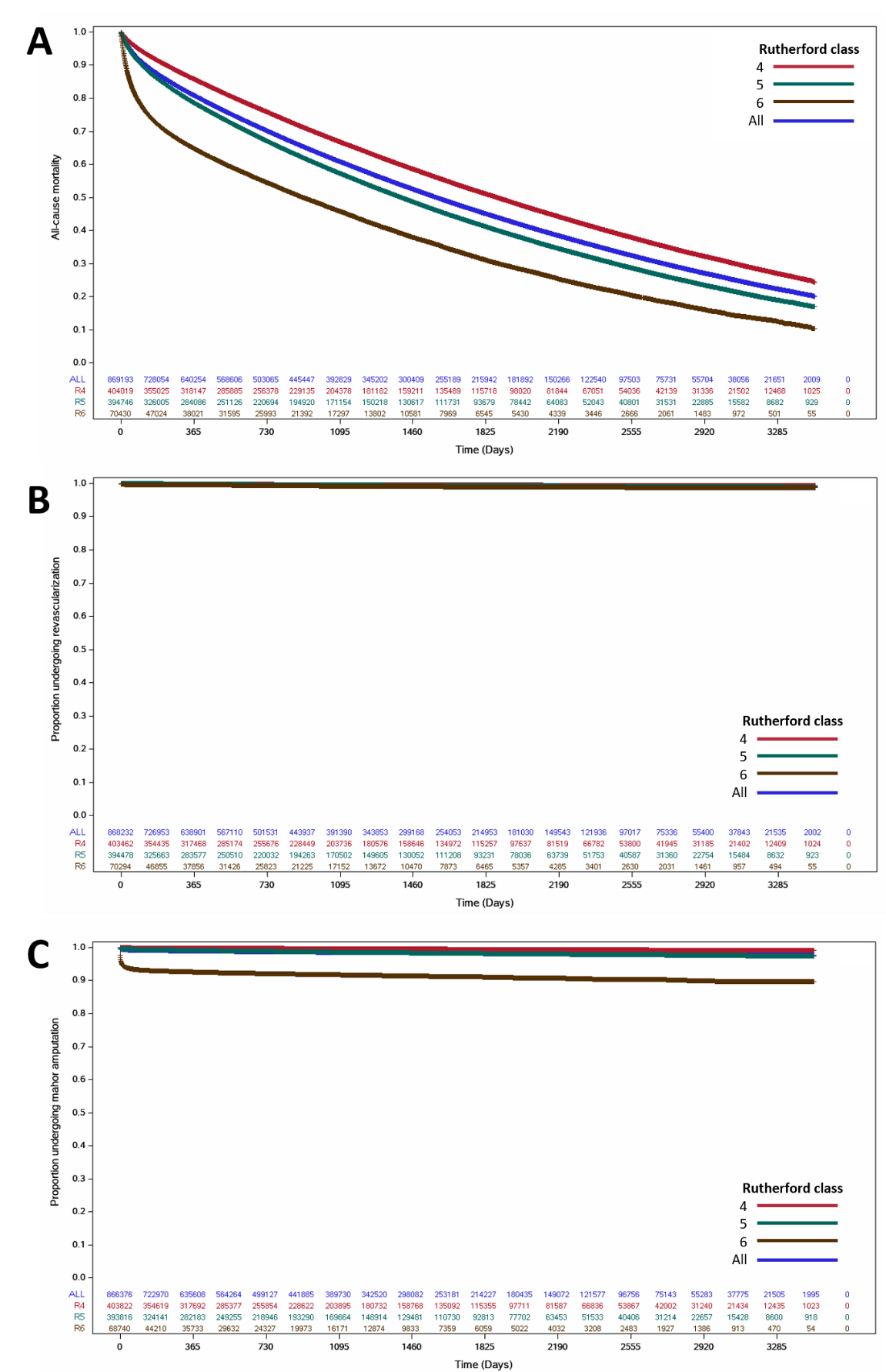
**

**Supplemental Figure 2:** Cumulative incidence of all-cause death (A), revascularization (B), and amputation (C) overall and by Rutherford class among patients with single CLTI-specific codes


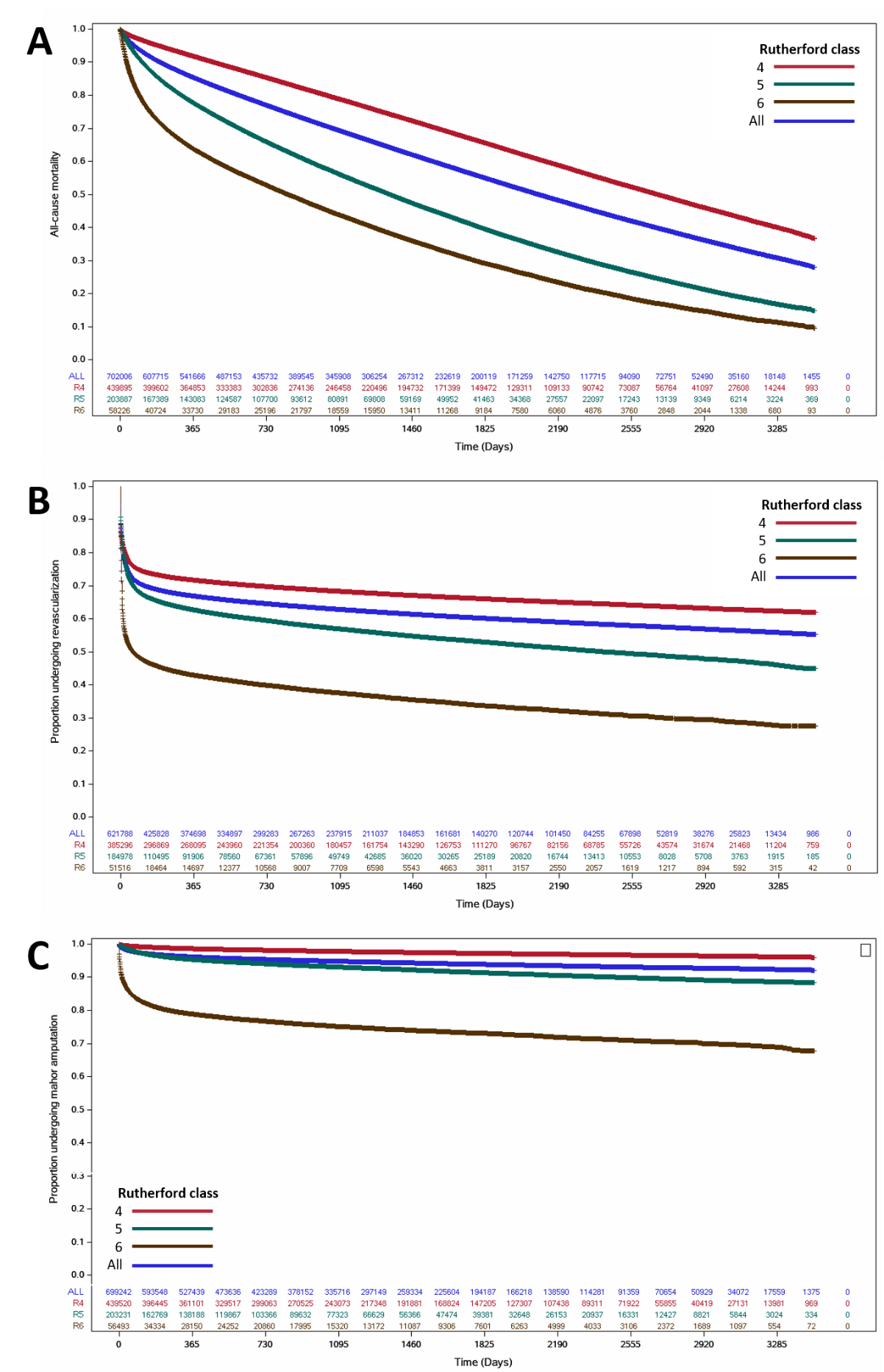

Supplement: Supplemental Materials [file mmc1.docx]
